# Integrating protein sequence design and evolutionary sequence conservation to uncover spectral tuning sites in red-light photoreceptors

**DOI:** 10.1016/j.str.2025.07.018

**Published:** 2025-11-06

**Authors:** Oliver Maximilian Eder, Massimo Gregorio Totaro, Stefan Minnich, Gustav Oberdorfer, Andreas Winkler

**Affiliations:** 1 Institute of Biochemistry, Graz University of Technology, Graz, Styria 8010, Austria; 2 BioTechMed-Graz, Graz, Austria

**Keywords:** phytochrome, bacteriophytochrome, ProteinMPNN, allosteric regulation, amino acid conservation, HDX-MS, structure function, spectral tuning, thermal reversion, phyB-4

## Abstract

Protein structure and function are defined by non-covalent interactions of the polypeptide backbone and amino acid side chains providing specific chemical environments. Understanding how these interactions impact stability and/or functional aspects of proteins is critical to understand fundamental mechanisms of life itself. However, assigning functional or structural roles to individual amino acids is challenging even if structural models are available. This study introduces the function-structure-adaptability (FSA) approach, a semi-automated pipeline leveraging evolutionary sequence conservation and ProteinMPNN to assign amino acid-level roles in proteins. Here, we show that the pipeline can identify previously undescribed functional allosteric regulation residues in a specific family of target proteins—red light-responsive phytochromes. Identified sites were targeted by amino acid substitution to explore their role in phytochromes spectral characteristics and thermal reversion properties. These results expand our understanding of the intricate regulation mechanisms in phytochromes. Furthermore, the FSA approach can be readily employed for other target proteins.

## Highlights

• Comparison of natural and ProteinMPNN sequences for structure-function assignment
• Discovery of allosteric network residues in the second shell cofactor environment
• Correlation of conformational dynamics with thermal reversion supported by HDX-MS
• Identification of tuning residues relevant for the whole phytochrome family

## Graphical abstract

**Figure:**
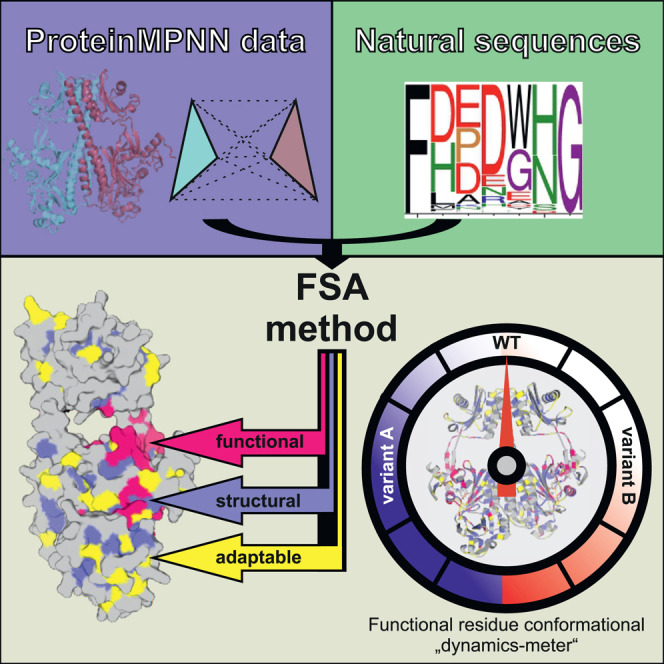

## Teaser

In this work, Eder et al. demonstrate that the structure-based design model ProteinMPNN can be used together with natural amino acid conservation to uncover functionally important residues in proteins. In a light sensing phytochrome, previously unknown functional residues were identified and their role was assessed by *in vitro* experiments.

## Introduction

Proteins are astonishing biological molecules responsible for a myriad of organismal functions, including the processing and generation of central metabolites, maintaining structural integrity, transporting molecules across membrane barriers, and integrating environmental stimuli. This diversity in functionalities is enabled by the evolutionary adaptation of the three-dimensional architecture of proteins while employing the same set of amino acid building blocks. Hence, the variable chemistries of specific amino acid side chains, the communication between secondary structure elements, and the intrinsic dynamics of protein structures are intricately linked to protein function.^1^^,^^2^

However, understanding and disentangling the deeply ingrained structure-function dependencies is often challenging, and even labor-intensive mutagenesis studies cannot always provide clear-cut answers. This is especially true because the fitness advantages needed for efficient deep mutational scanning approaches^3^ are not always easily linked to the diversity of protein functionalities of interest. More traditionally, data from evolutionary sequence conservation and structural models are used to pre-filter important amino acids suspected to be involved in protein functionality. More recently, co-evolutionary analyses^4^ have also helped to identify important non-obvious interactions. These early computational pipelines performed relatively well; however, a sufficiently large protein sequence dataset was pivotal for extracting meaningful annotation of residues. Another general downside of these methods is that amino acid conservation and co-evolution represent a continuous spectrum between the extremes of purely functional roles of a residue—for example ligand coordination—to contributions to protein folding and structural stability.^5^ Lately, the utilization of huge protein sequence datasets led to the incorporation of generalizable protein folding rules into computational pipelines. This enabled the emergence of powerful neuronal networks for residue-level categorization like ESM-scan,^6^ ProGen,^7^ RXNAAMAPPER pipeline,^8^ and AlphaMissense.^9^ While these approaches still fail to differentiate between functional and structural stability properties, recent work has endeavored to include protein stability effects through machine learning models informed by thermodynamic considerations.^5^

In this work, we introduce the function-structure-adaptability (FSA) workflow which repurposes existing machine learning models and evolutionary residue conservation to distinguish functional and structural roles of residues. Unlike previous approaches, FSA employs a distinct methodology that outperforms the tool presented by Cagiada et al.,^5^ in our benchmarking protein. Central to the FSA approach is a statistical pipeline that compares curated multiple sequence alignments (MSAs) of natural protein sequences with those of ProteinMPNN output sequences.^10^ The latter tool is a deep learning model allowing the generation of novel protein sequences retaining the fold of an input backbone structure^11^^,^^12^ that can derive from data of either X-ray crystallography, cryoelectron microscopy (cryo-EM), NMR, or accurate structure model generators, like AlphaFold2.^13^^,^^14^ Since ProteinMPNN generates “idealized” sequences for the target protein backbone, deviations from this idealization in natural sequences may indicate functionally relevant residues. On the other hand, high conservation of residues in both natural sequences and MPNN runs might support a more structural role of the corresponding position.

**Figure 1:**
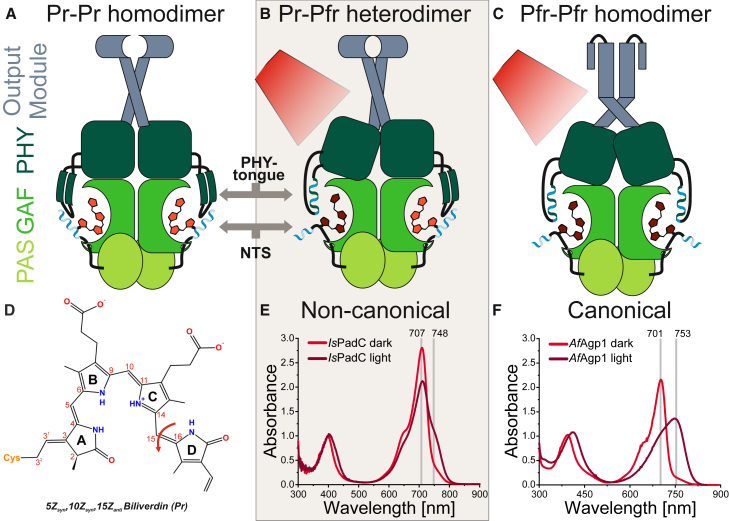
Domain arrangement and central photochemical properties of bacteriophytochromes The prototypical PAS (grass green)-GAF (green)-PHY (dark green)-output module (grey-blue) arrangement of bacteriophytochromes is depicted. The N-terminal segment (NTS, blue) and the PHY tongue (dark green β sheets or blue-green helix) are shown in their light state-dependent conformations. The BV cofactor is colored in orange-red (Pr state) or brown-red (Pfr state). (A) Prototypical Pr resting state. (B) Red light-illuminated non-canonical activated state (*Is*PadC model) featuring an asymmetric Pr-Pfr heterodimer assembly. (C) Red light-illuminated canonical activated state (*Af*Agp1 model) featuring a Pfr-Pfr homodimer assembly. (D) Chemical structure of the BV cofactor covalently bound to BphPs. Red light isomerizes the C15 = C16 double bond, causing a D-ring flip and stabilization of the 15*E* configuration by surrounding residues in Pfr. (E) Spectral characteristics of a non-canonical model phytochrome (*Is*PadC) with Pr (red) and illuminated state trace (brown-red) with incomplete conversion to Pfr. (F) UV-vis spectrum of a canonical model phytochrome (*Af*Agp1; data from ref.^19^) with more complete Pfr formation.

To address the potential and limitations of the approach outlined previously, we selected a model protein of allosterically regulated red light-sensitive phytochromes.^15^ This protein family proved difficult to analyze by existing methods, especially due to its underlying complex allosteric tuning mechanisms. Generally, these multi-domain proteins represent red/far-red light switchable photoreceptors occurring in plants, algae, bacteria, and fungi.^16^ They play a pivotal role in integrating a key actuator for living organisms—the presence or absence of ambient light. The subfamily of bacteriophytochromes (BphPs) is characterized by an interesting modularity of covalently linked effector domains of which several have recently been characterized in detail.^17^ Hallmarks of BphPs are biliverdin (BV) as the light-sensing cofactor covalently linked to a cysteine of the N-terminal segment (NTS)^18^ and a PAS (Period/ARNT/single-minded), GAF (cGMP phosphodiesterase/adenylyl cyclase/FhlA), PHY (phytochrome specific) three-domain architecture, typically in a parallel dimeric arrangement (Figures 1A–1C).^15^

The key to light signal integration is the BV cofactor and its embedding in the GAF domain which provides the chemical environment that stabilizes the linear tetrapyrrole cofactor.^15^ Completing the BV binding pocket, a protrusion of the PHY domain, the so-called PHY tongue, and the NTS helix both shield the cofactor from solvent.^20^^,^^21^ Upon light activation, BV isomerizes and thereby flips the tetrapyrrole D-ring (Figure 1D). This results in side-chain rearrangements in the cofactor’s environment and, via a series of structural intermediates involving altered hydrogen bonding interactions,^22^^,^^23^ in the adaptation of PHY tongue and NTS conformations. These are characterized by a β-hairpin to α helix transition and a repositioning of the chromophore-attached NTS helix (Figures 1A–1C), respectively.^21^^,^^24^^,^^25^ In prototypical phytochromes the resulting Pfr conformation (phytochrome absorbing far-red) is a meta-stable ground state that either thermally or by far-red illumination reverts to the dark-adapted Pr state (phytochrome absorbing red).^16^ However, many phytochromes feature photostationary states (PSSs) showing indications of Pr, Pfr, and/or intermediate species (Figure 1E), and hence their incomplete Pfr formation is sometimes referred to as non-canonical behavior.^26^^,^^27^ Considering the frequent description of structural asymmetry in the dimeric structures of phytochromes,^28^^,^^29^ the stabilization of non-canonical states might actually be of functional relevance.^30^^,^^31^

Applying the FSA approach to the family of phytochromes, we compared *in silico* results for two proteins, *Af*Agp1 and *Is*PadC. The former is a model protein belonging to the well-characterized histidine kinase (HK)-linked prototypical BphP family. *Is*PadC, on the other hand, is a representative of GGDEF-linked phytochromes that mostly feature non-canonical members.^32^ Initial FSA annotation results were benchmarked using the wealth of functional annotations available for the HK-subfamily.^33^^,^^34^ Thereby, intriguing differences between the HK and GGDEF model proteins became apparent, which we sought to address experimentally as part of this study for the model protein *Is*PadC. By leveraging the FSA method, a protein region capable of tuning the phytochrome conformational landscape and thermal reversion properties could be identified as determined by UV/vis experiments and hydrogen-deuterium exchange coupled to mass spectrometry (HDX-MS).

Overall, these findings expand our understanding of the intricate allosteric tuning networks in BphPs, also with implications for the related and very important plant phytochromes. Generally, the FSA method is a valuable addition to the toolbox of protein annotation methods that is also applicable to other protein families. Thereby, the FSA method could help to further our understanding of protein regulation networks not only relevant for basic research, but also for therapeutic proteins and industrial applications.

## Results

### Workflow and class definition of the FSA approach

We established the FSA approach to identify functional and structural residues in proteins and tested it on our model protein for allosteric regulation—the red light sensitive phytochrome *Is*PadC. By filtering out conserved residues assigned as structural, we can focus on functional amino acids involved in modulating the photocycle properties of this protein. As this can readily be tested by a spectral characterization of protein variants, the FSA analysis was restricted to the photo sensory module (PSM) omitting the enzymatic domain to streamline computational workflows. To narrow down the functional positions proposed by the pipeline, the FSA-pattern of *Is*PadC was compared to the annotation of a member of the phytochrome-HK family (*Af*Agp1), trying to pinpoint regions responsible for the distinct photocycle characteristics of the two homologs (Figures 1E and 1F).

**Figure 2:**
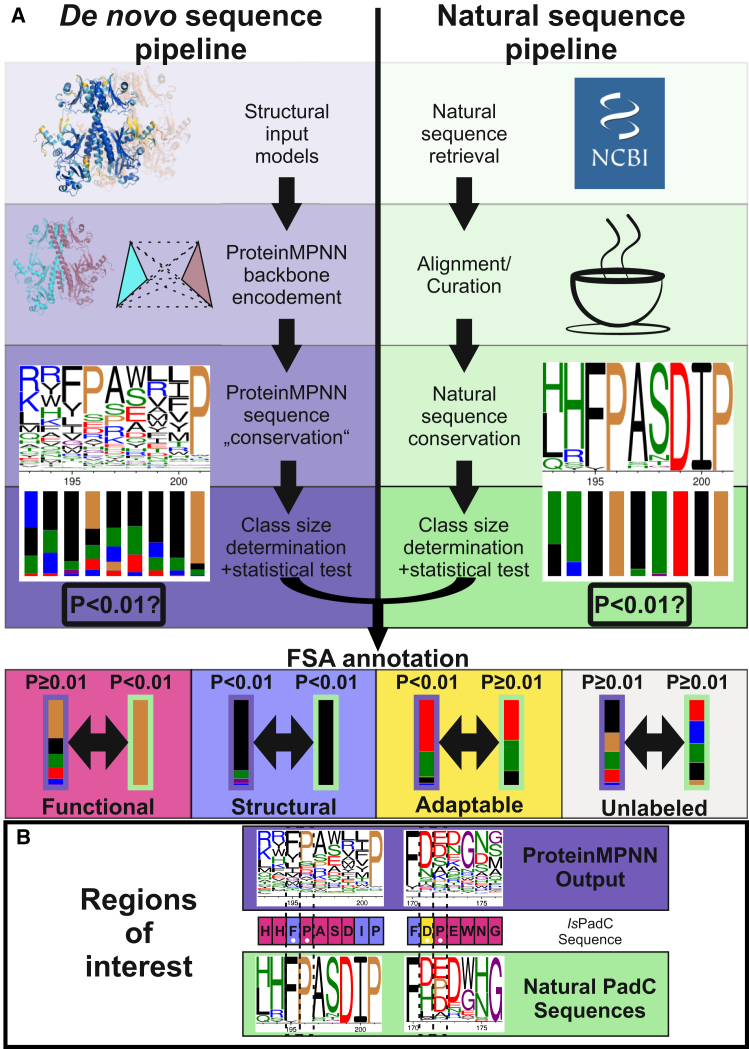
Overview of the FSA pipeline and example output (A) Workflow for generating ProteinMPNN sequences and retrieving/curating natural protein sequences. For the neural network-based workflow (left), multiple structural models of a target protein are provided to ProteinMPNN. Naturally occurring sequences are retrieved from repositories (right) and are aligned and curated using T-coffee and Jalview. For both datasets, position-specific scoring matrices (PSSM) are generated to represent sequence conservation. Amino acids are grouped according to chemical properties (see text) and the size of each group is calculated for each position. A *p* value testing the significance of class enrichment is assigned to each position. For FSA annotation, the *p* values of ProteinMPNN and natural sequences are compared. The four possibilities lead to the classifications functional, structural, adaptable or unlabeled. (B) FSA annotation for selected regions of interest in the protein of this study - *Is*PadC. The multiple sequence alignments of ProteinMPNN output sequences (blue background) and natural PadC sequences (green background) are shown for the PASDIP motif and a turn region discussed below. Pro196, Phe195, and Asp171 are marked with a white circle and dotted lines as they reflect the “FSA annotation” examples of the panel above. Pro172 is highlighted as it represents a special case of annotation as outlined in the discussion. Weblogo colors depict the chemical classes of amino acids as follows: hydrophobic residues (black), negatively charged residues (cherry red), positively charged residues (dark blue), polar residues (green), glycine (purple), proline (brown). Also see Figures S1–S4.

The general workflow for the FSA pipeline is depicted in Figure 2A. On one side, the approach is leveraging ProteinMPNN’s ability to design novel amino acid sequences that structurally recapitulate an input structure. For this purpose, structural model coordinates were provided to ProteinMPNN to generate 1,000 novel output sequences. To limit model bias and prevent skewed residue selection due to model inaccuracies or artifacts, ProteinMPNN was run in the multistate-design approach.^10^ Hence, ProteinMPNN was provided with three different structural models of the same protein. For example, in the case of *Is*PadC, the highest-ranked relaxed Alphafold2 prediction and two crystal structures from different crystallization conditions (PDB: 5llw, 5lly) were used. The network then predicted residues best fitting into each sequence position, effectively averaging over the three structures. In parallel, naturally occurring bacteriophytochrome sequences were retrieved from databases and filtered to ensure the presence of a BV binding Cys^BV^ and the same output domain (GGDEF or HK) as minimal requirements.

The retrieved natural and ProteinMPNN generated sequences were then aligned and subjected to analysis by position-specific iterated (PSI)-BLAST generating position-specific scoring matrices (PSSMs) as outputs (Weblogo depictions of *Is*PadC and *Af*Agp1 alignments in Figures S1–S4). A robust, iteratively optimized method was then developed to analyze amino acid enrichment patterns in the PSSMs (details see STAR Methods section). In brief, amino acids were grouped by chemical properties like hydrophobicity, polarity, or charge. A statistical test (Mann-Whitney U, *p* = 0.01) was used to uncover significant enrichment of amino acid classes for each position by independently analyzing the natural and computer designed sequences. Subsequently, enrichment patterns were compared between natural and non-natural sequences. This side-by-side evaluation revealed three distinctive enrichment patterns. In class 1, amino acids are highly conserved in the naturally occurring sequences, whereas the corresponding position lacks any preference in the ProteinMPNN output. In class 2, both evolution and the deep learning approach converge to the same class of amino acids in the same position. In class 3, there is a high diversity of amino acids in natural sequences but a clear preference in ProteinMPNN sequences.

**Figure 3:**
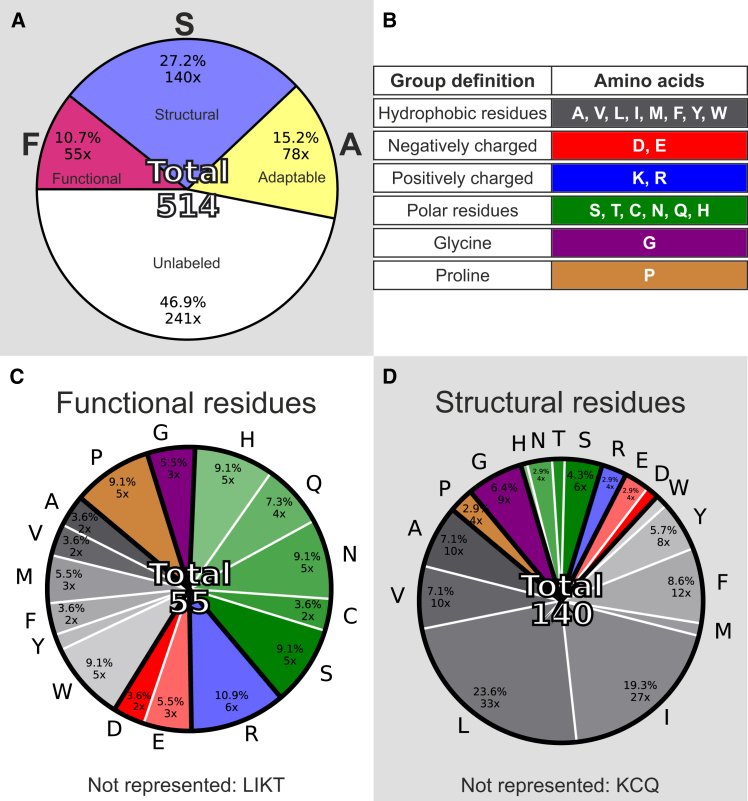
Statistics of the FSA analysis conducted on *Is*PadC (A) Percentages of each class’ assignments. Of the 514 amino acids in the photosensory module, 10.7% are flagged as functional (pink), 27.2% as structural (metallic blue), 15.2% as adaptable (yellow), and 46.9% are not flagged (white). (B) Grouping of amino acids according to physico-chemical properties as outlined. (C and D) Amino acid composition of the assigned classes for functional and structural residues, respectively. Amino acid frequencies are depicted as percentages of the total number shown in the middle of the pie chart and as absolute numbers (e.g., 5×). Percentage values lower than 2% are not displayed in the figure. Also see Figure S5.

**Figure 4:**
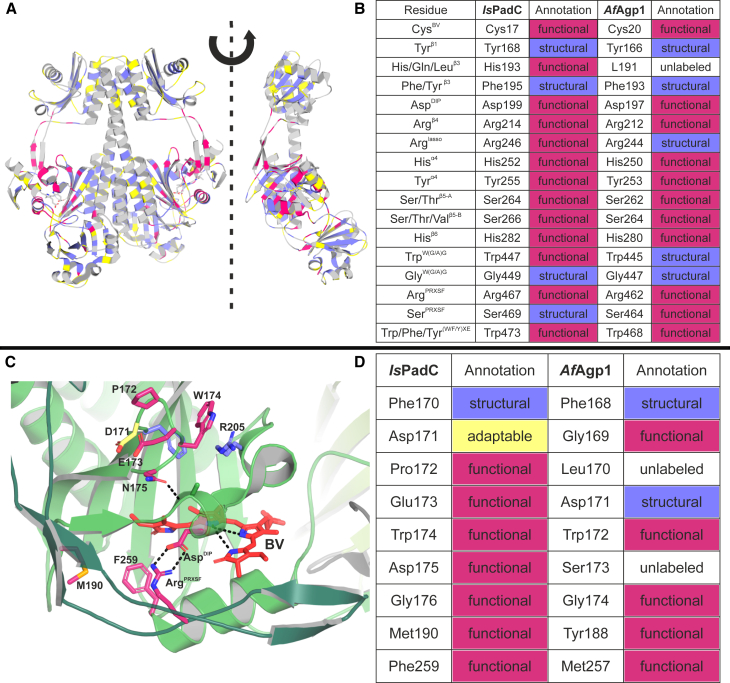
Visualization and validation of the FSA results (A) “Functional” (pink), “Structural” (metallic blue), and “Adaptable” (yellow) annotations plotted on the phytochrome dimer in front view (left) and of a rotated monomer in side view (right). (B) Validation of the FSA workflow results against the published list of key functional residues of phytochromes.^15^ (C) *Is*PadC Pr structure (PDB: 5llw) shown with selected BV second-shell residues (sticks) of the GAF domain (green cartoon). Sidechains in the turn region 170–176, Met190, and Phe259 are highlighted according to their FSA classes. Note that Trp174 (“functional”) is stacking with Arg205 (“structural”) while Asn175 (“functional”) forms a hydrogen bond with an amide-proton of the helix containing Asp^DIP^ within the PASDIP motif. In addition, the BV cofactor and PHY tongue are shown in red and dark green, respectively, and the pivotal Pr interaction between Arg^PRXSF^ and Asp^DIP^ is highlighted (dotted black lines). The N-terminal segment is not shown to aid clarity. (D) FSA class comparison of the turn region between *Is*PadC and *Af*Agp1. Also see Figures S6 and S11.

Apparently, in class 1, the chemical nature of residues is deemed essential by nature but is ignored by ProteinMPNN as their side chains might not be critical for the fold or stability of the protein. A pivotal residue flagged as class 1 is Asp^DIP^, a residue which shows strict conservation by nature but is ignored by ProteinMPNN (nomenclature from the study by Hughes and Winkler,^15^ Asp199 in *Is*PadC). However, also less strictly conserved positions can be considered when the sum of amino acids belonging to the same chemical group (defined in Figure 3B) clears a certain threshold. For example, adding up the conservation of histidine and glutamine in position 193 suggests that polar residues are conserved in the natural sequences. Since the ProteinMPNN sequence conservation does not show any significant enrichment of a chemical group, *Is*PadC His193 is also assigned to class 1 (Figure 2B). We suggest that positions with conservation patterns in natural sequences, but not in ProteinMPNN, are classified as “functional”. This “functional” subcategory consists predominantly of polar residues (total 38.2%) like serine and histidine as well as charged residues like aspartate and arginine (total 20%) as depicted in Figure 3C. Noticeably, hydrophobic residues are underrepresented when compared to the overall abundance of amino acids in the *Is*PadC PSM sequence. Tryptophan, however, poses an exception to this trend as the observed enrichment likely reflects its unique chemical and structural properties in specific protein environments. Examples of functional tryptophans are Trp447 and Trp 473, which are known to constitute the so-called Trp switch, an essential element for light sensing in phytochromes.^20^ Overall, the visualization of residues annotated as functional on the respective phytochrome structures (Figure 4A) revealed that they cluster in the BV binding pocket, the PHY tongue, and stretches of amino acids forming the characteristic phytochrome knot.^35^ These structural elements confer light-sensing capabilities to phytochromes and are deeply connected to the inherent function of this protein family, which justifies their categorization as “functional”.

In contrast, class 2 residues are conserved in both natural and ProteinMPNN sequences. An example for this pattern is Phe195 which is deemed essential both by nature and the neural network (Figures 2A and 2B). In this class, aromatic residues like phenylalanine, tyrosine, and other hydrophobic core packing residues like leucine are overrepresented compared to the overall abundance of amino acids in the *Is*PadC PSM sequence (Figure 3D). However, also glycine and proline are frequently assigned to this class. This underlines that this category detects hydrophobic core packing side chains *per se* but also backbone geometry requirements for correct folding. Visualizing class 2 residues on a phytochrome structure, we observed a tendency for clustering at the core of protein domains (Figure 4A). Overall, this led to the designation of this class as “structural” residues.

Somewhat unexpectedly, we also identified a third class of “adaptable” residues (class 3). In this class, ProteinMPNN prefers a subset of amino acids without conservation in the evolutionary sequences. However, exploring the implications of this category was designated beyond the scope of this study; especially considering the strong influence of subtle input model geometry differences on this class as well as its stronger response to altering threshold values in the FSA workflow. Still, hypotheses and implications surrounding this group are provided in the discussion and more in-depth visualizations are shown in Figure S5.

In the context of cofactor-binding proteins, it should be mentioned that ProteinMPNN was recently extended to also handle protein-ligand interactions. Instead of designing protein sequences in isolation, LigandMPNN^36^ incorporates ligand binding information to generate sequences optimized for binding affinity and structural compatibility with potential ligands. We also ran the FSA workflow using LigandMPNN and a set of three structural models including the BV cofactor (two crystals structures and one Chai server prediction^37^). The results shown in Figure S6 demonstrate that several residues interacting with the cofactor change their classification indicating the awareness of LigandMPNN for the included cofactor. In fact, direct cofactor interacting residues are preserved by LigandMPNN leading to a change in their classification from functional to structural. Still, the majority of functional assignments remain unaltered and the other categories seem to be more sensitive to the usage of ProteinMPNN or LigandMPNN, putatively due to differences in network training and input structures. Since we consider chromophore binding and structural rearrangements in its environment a function of the photoreceptor, we focused on the results of the FSA workflow using ProteinMPNN in the remaining manuscript. Nevertheless, it should be emphasized that different input structures and different MPNN versions can result in altered assignments, especially in cases where the statistical test is borderline, as observed for some residues assigned as structural or adaptable.

Benchmarking of the FSA pipeline performance was conducted by assessing its ability to identify first-shell key residues that directly contact BV (Figure 4B), as their properties have been described extensively in the literature for model phytochromes like *Af*Agp1 and *Dr*BphP.^33^^,^^34^ Assessment of the FSA-assigned classes for the *Is*PadC and *Af*Agp1 sequences revealed that most of the residues interacting with BV are flagged. While 13 and 11 positions are flagged as “functional” in *Is*PadC and *Af*Agp1, respectively, some residues are also categorized “structural” as depicted in Figure 4. More details regarding the flagging as either functional or structural and the slight differences in flagging patterns between the homologs are provided in the discussion.

Assessing the global FSA annotation patterns between *Is*PadC and *Af*Agp1, the pipeline suggests differences between these distantly related phytochromes. By experimentally addressing such characteristic “functional” residues, which are also not yet described in the literature, we aimed to discover protein regions that contribute to the characteristic spectral differences between the two branches of BphPs. Testing the specific influence of individual positions is frequently not straight-forward due to co-evolutionary interactions and/or secondary effects upon amino acid replacements. For this study, we chose a combination of rationally considering the intended effects on characteristic interactions and/or the enrichment of other amino acids in the alignment of the respective positions. In addition, we pre-assessed the feasibility of substitutions using ESM-scan.^6^ In the context of spectral properties, positions which do not directly contact the BV cofactor, i.e., second-shell residues that also show variation between the homologs were deemed promising, as these positions remain an underexplored area in phytochrome research. Additionally, an interesting couple of functionally flagged residues which directly connect the second-shell environment with the BV D-ring in *Is*PadC, was probed in greater detail.

### Addressing the connection between the BV environment and second-shell residues

Two residues that were not described as coupled positions in the literature but flagged as “functional” by FSA in *Af*Agp1 and *Is*PadC, are Tyr188/Met190 and Met257/Phe259, respectively. The apparent swap of an aromatic amino acid with methionine stands out in *Is*PadC, while most other bacteriophytochromes feature an *Af*Agp1-like arrangement. Noticeably, these residues are in proximity of the BV D-ring and the PHY-tongue region, thereby potentially influencing D-ring flipping and Pfr stabilization (Figure 4C). In addition, Tyr188/Met190 is part of a second-shell structural element that was previously suggested to influence the spectral properties of BphPs like RpBphP3^38^ and *Is*PadC.^27^ To investigate this further, we generated an *Is*PadC PSM M190Y/F259M variant and assessed the impact of this exchange on spectral properties.

**Figure 5:**
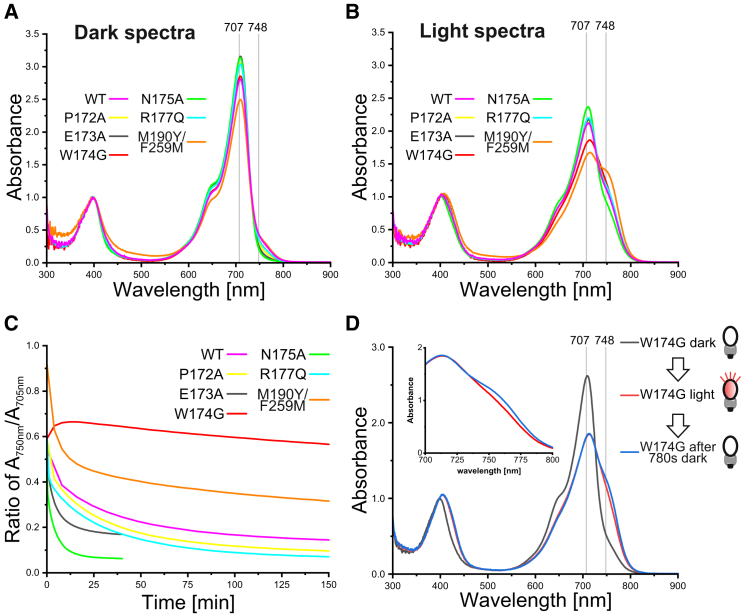
UV/vis spectra and thermal reversion characteristics of *Is*PadC PSM variants (A and B) Pr and photostationary state (PSS^660nm^) spectra of characterized second-shell substitution variants, respectively. The wild-type (WT) absorbance spectrum is shown as reference (pink). Gray vertical lines highlight the minimum and maximum of the WT difference spectrum (PSS^660nm^ minus Pr). All traces were scaled to 1 according to their Pr 398 nm Soret band maximum. (C) Thermal reversion behavior of *Is*PadC PSM WT and variants. The 750/705 nm absorbance ratio is plotted against the recovery time. (D) Spectral details of the unusual temporal behavior of the *Is*PadC PSM W174G variant shown in (C). Its Pfr shoulder increases for 780 s (blue trace) after switching off the light to record the PSS660 (red trace). A close-up of the relevant region is shown in the inset. Also see Figures S7 and S8.

Intriguingly, this double variant exhibited the strongest shift toward Pfr among any *Is*PadC variant characterized so far (Figures 5B and S7). Nevertheless, no full Pfr is formed and the thermal reversion from the photostationary state still shows biphasic characteristics indicative of a faster recovering Pfr/Pfr population and a more stable mixed Pfr/Pr population. Analysis of individual variants revealed that F259M already enables increased Pfr formation, while M190Y with two aromatic residues close in space has a detrimental effect on Pfr formation (Figure S7). The latter observation demonstrates that even second-shell residues can exhibit substantial effects on the spectral properties.

### Second-shell variants influence spectral properties and thermal reversion in phytochromes

Prompted by the pronounced effects observed for cofactor-distant residues aforementioned, we inspected another second-shell region around the BV cofactor in *Is*PadC. The turn between strands β1 and β2 of the GAF domain (residues 170–175 in *Is*PadC) is more than 10 Å away from the cofactor, and its side chains were predominantly flagged as “functional” by the FSA approach (Figure 4D). Interestingly, the FSA annotations of the corresponding residues in *Af*Agp1 deviate substantially from those of *Is*PadC, which suggests potential functional differences of this turn element. To test this hypothesis, we substituted each turn position individually in the *Is*PadC photosensory module (PSM). The P172A variant was designed to disrupt the stabilizing effects of proline in the middle of the turn, while E173A targeted potential ionic interactions with nearby residues. W174G was particularly intriguing, as over 80% of natural PadC sequences feature either tryptophan or glycine at this position, two amino acids with strongly diverging properties. In the *Is*PadC structure (PDB: 5llw), Trp174 forms a prominent cation-π-stacking interaction with Arg205, which would be lost when substituted with glycine. Additionally, N175A was introduced to disrupt the unique hydrogen bond between the asparagine amide group and the PASDIP motif, a region critical to phytochrome photochromicity.

To assess effects beyond flagged residues, the variant R177Q was created to probe an unflagged residue near the β-turn 170–175. All variants, including R177Q, were successfully overexpressed and purified with high yields for initial UV/Vis and thermal reversion characterization. In addition, the role of Asp171, annotated as “adaptable”, was investigated by substituting it with leucine; however, no soluble protein could be obtained.

Intriguingly, the variants showed either no or only minute changes in their UV/vis traces in the Pr or in the PSS^660nm^ spectra (Figures 5A, 5B, and S7). Additionally, in the thermal reversion experiments, both the control variant R177Q and P172A displayed WT-like behavior. In stark contrast, thermal reversion was strongly altered in the other protein variants (Figure 5C). N175A and E173A showed a roughly 10× and 5× accelerated thermal reversion rate, respectively, compared to that of the wildtype. Strikingly, variant W174G featured an extremely slow thermal reversion as it failed to fully revert to Pr even after 24 h. This is remarkable, since position 174 is flanked on both sides by inverse behaving positions. Additionally, illuminating W174G with 660 nm light uncovered an unusual light cycle behavior. Initially, red light populated a typical non-canonical steady-state light spectrum (Figure 5D, red trace). Yet, quite unexpectedly, upon switching off the light source, the Pfr shoulder became even more pronounced (Figure 5D, blue trace) before classical thermal reversion characteristics took over around 780 s. This effect is also visible in Figure 5C, where the A_750nm_/A_705nm_ trace for W174G initially increases. Therefore, central aspects of the phytochrome photocycle, such as short-lived intermediates and/or efficient progression through the photocycle, seem to be noticeably affected by the W174G substitution.

The reason behind the drastic effects of the single amino acid substitution W174G on the thermal reversion and photocycle properties is not evident from the static crystal structures. To further investigate this striking behavior, the *Is*PadC PSM WT was assessed as a reference and the variant N175A was included due to its opposite effect albeit neighboring position. In fluorescence measurements, no substantially altered emission or excitation characteristics of W174G or N175A compared to the wildtype could be observed (Figure S8). Hence, substantial radiative off-pathways in parallel to the photocycle were excluded. It was hypothesized that substitution-induced changes in the overall conformational dynamics might contribute to the observed effects, as in W174G and N175A, a stabilizing cation-π-stacking interaction and a hydrogen bond are eliminated, respectively. To address this hypothesis in detail, we employed HDX-MS to gain peptide-level resolution of conformational dynamics in the variants.

### HDX-MS reveals strongly altered protein dynamics for W174G and N175A

**Figure 6:**
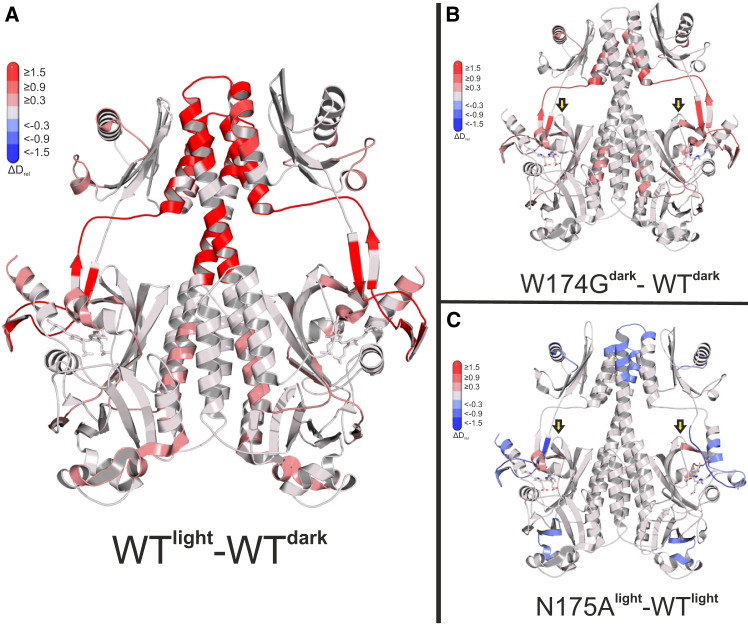
HDX-MS characterization of *Is*PadC PSM in dark and light states (A) Differences in deuterium exchange (ΔD_rel_) of WT^light^ minus WT^dark^ datasets after 30 s of deuterium incorporation mapped onto the structure of *Is*PadC. (B and C) Relative deuterium uptake of the indicated variants versus the respective WT datasets (ΔD_rel_ = D_variant_ - D_WT_). (B) compares the 10 s time point of W174G (dark-adapted) and (C) compares the 3 min time point of N175A (red light illuminated). The respective light and dark datasets are provided in Figure S10. The positions targeted by substitutions are highlighted by yellow arrows. Red or blue regions, according to the bar legend, highlight increased or reduced deuterium incorporation in the target versus the reference state, respectively. Dark adapted and red light-illuminated structures (PDB: 5llw, 6et7) were used as models for visualization, respectively. Also see Figures S9 and S10.

It was previously shown that different phytochrome photo-states are accompanied by pronounced differences in conformational dynamics.^27^^,^^39^^,^^40^^,^^41^ The presented *Is*PadC PSM variants mirror this behavior as the PSS^660nm^ resulted in increased dynamics relative to the dark state in the PHY tongue, the NTS, and the GAF dimer interface. Additional less affected structural elements are the PASDIP motif, the GAF lasso, and parts of the PAS domain (Figure 6A). As a side note, the *Is*PadC PSM C-terminal α helix seemingly acquired more degrees of freedom compared to the full-length context, likely due to the removal of the enzymatic domain. To examine the effects of the variants W174G and N175A, dark and light HDX-MS datasets were compared with those of the WT reference (Figures 6B, 6C, S9, and S10).

Especially the W174G variant already exhibits increased dynamics in the aforementioned characteristic light-responsive regions in the dark state (Figure 6B). The opposite behavior is observed for N175A where the light dataset showed pronounced changes with reduced deuterium incorporation especially in the PAS domain and PHY tongue peptides.

For peptides containing the W174G substitution, deuterium uptake increased only slightly compared to the low rates in the wildtype (Figure S9A). Since the Trp174 containing turn element appears to be embedded in the core structure of the GAF domain, also the direct vicinity of its substitution is not strongly affected. However, eliminating the interaction of Trp174 with Arg205 leads to increased dark state dynamics in the PASDIP region (191–204), a key motif for proper cofactor coordination and light sensing (Figure S9B). Similarly, removing the direct backbone interaction with the PASDIP motif in variant N175A also causes increased conformational dynamics for these peptides for intermediate timescales of deuterium exchange (Figure S9B). Remarkably, these limited local changes in dynamics propagate throughout the entire protein and have a strong influence on distant regions, some of which are responsible for light sensing.

But how does this relate to the drastic differences in thermal reversion? Looking at the deuterium incorporation in more detail revealed that another central phytochrome element, the WAG motif in the PHY-tongue (Figure S9C), shows an unexpected bimodal characteristic only for W174G. Already in the dark dataset, W174G features two separate conformational substates of the PHY tongue conformation: one resembling WT^dark^ and the other WT^light^-like behavior (Figure S5D). Therefore, the driving force to return to the Pr-inhibited β-hairpin conformation appears to be reduced in W174G, which adds an additional explanation for the increased lifetime of the alpha-helical Pfr contribution where the WAG motif is typically disordered.^15^ Summing up, a remarkable example for allosteric regulation was uncovered where minute local changes triggered by single substitutions override the finely tuned energetic landscape of the photoreceptor *Is*PadC.

## Discussion

### Identification of functionally important residues in phytochromes

Even in the era of high-accuracy structure prediction tools like Alphafold2/3,^13^^,^^42^ ESM,^43^ or Chai,^37^ deriving protein sites critical for functionality solely from a 3D model is challenging. While amino acid conservation and co-evolution can be an initial indication, it is often difficult to determine whether identified residues contribute primarily to functional or structural aspects of the protein. This is especially true for regions that fine-tune properties in a rheostat-like^44^ manner where the conservation signal may be more diverse than in residues essential for particular functions, for example ligand coordination. The FSA pipeline described in this study allows the identification of important residues and their categorization into functionally or structurally relevant amino acids. In our specific test case, this led to the discovery of previously undescribed positions that tune photochromicity and thermal reversion behavior in *Is*PadC. Therefore, even with constantly improving deep mutational scanning strategies and the availability of high-throughput techniques, prefiltering for first line target residues can be of importance in systems where no obvious fitness advantage can be used to screen for improved properties.

Among the functionally flagged residues, the Met190-Phe259 pair, which is unique to *Is*PadC and one close homolog, was targeted and exchanged to the context predominantly found in phytochrome family members. In the resulting *Is*PadC PSM variant M190Y/F259M, a significantly higher proportion of Pfr was stabilized in the photostationary state upon red-light illumination than in the wildtype. While this variant still failed to feature a full Pfr spectrum, it represents the furthest shift toward this direction for *Is*PadC. Interestingly, only one other phytochrome in the PDB lacks an aromatic residue at the position corresponding to Met190 in *Is*PadC, the unusual non-shifting *Rp*BphP3. Mutagenesis studies on *Rp*BphP3 Leu207 and *Rp*BphP2 Tyr193 (both corresponding to Met190 in *Is*PadC) uncovered rather strong effects of substitutions in this position on the absorption spectra and thermal reversion behavior.^38^ This is in line with the results of the presented study, indicating an involvement of second-shell residues distant from the cofactor in light sensing. Concerning position Phe259, a unique π-stacking interaction with Phe^PRXSF^ was observed in the Pr structure of *Is*PadC (PDB: 5llw). Upon illumination, this interaction might either disfavor Pfr formation in the wild type or stabilize the Pr conformation, as the single-variant F259M already features a higher proportion of Pfr in the photostationary state. In summary, the described amino acid switch provides an elegant fine-tuning solution to *Is*PadC’s specific functional requirements.^26^

Using the FSA pipeline, we also identified a hotspot of tuning residues located on a turn between GAF β1 and β2 that affect thermal reversion. Unexpectedly, targeting this region by substituting N175 for an alanine (N175A) significantly accelerated the thermal reversion rate, while substituting the adjacent residue W174 for a glycine (W174G) substantially decelerated the thermal reversion. In addition, variant W174G showed atypical photocycle behavior characterized by a substantially slowed progression through photocycle intermediates. These contrasting effects were caused by the disruption of interactions in the second shell of the BV coordinating residues. Emanating from a singular substitution point, altered conformational dynamics landscapes of the two variants, as uncovered by HDX-MS analyses,^45^ impact Pr and Pfr stabilities differently.

Notably, conformational dynamics and thermal reversion rates of photoreceptors are tightly correlated. While this was shown specifically for *Is*PadC and variants stabilizing its Pfr state recently,^27^ also indirect evidence exists from other phytochrome studies. For example, the removal of the output module in *Dr*BphP-PSM was associated with increased conformational dynamics in the tongue region by FTIR.^46^ In addition to altered dynamics, *Dr*BphP-PSM exhibited substantially slower thermal reversion than the full-length construct.^47^ In this light, the current study proposes the turn region between GAF β1 and β2 (residues 170–175 containing Trp174 and Asn175 in *Is*PadC) as another important region capable of tuning spectral properties by modulating conformational dynamics. However, the question remains: are these implications generalizable to other homologs proteins?

Hints that effects like those triggered by N175A are generalizable across the phytochrome superfamily arise from mutagenesis studies of *A. thaliana* phyB variants. There, the H283Y substitution—corresponding to position Asn175 in *Is*PadC—resulted in phenotypes such as elongated petioles and early flowering.^48^ Biochemical characterization correlated H283Y (phenotype also called phyB-4 or hy3-4 117) with reduced Pfr stabilization and a moderately accelerated (∼50%) thermal reversion rate.^49^ These effects resemble those observed for N175A in *Is*PadC in the present study and imply that the discussed β-turn is a tuning element across all phytochrome families. Further investigation of substitutions at positions homologs to Trp174 and Asn175 might reveal additional phenotypes linked to partially altered light sensing and might expand our understanding of regulatory networks in phytochromes across different kingdoms of life.

In bacteriophytochromes, the uncovered β-turn region exhibits relatively high sequence diversity. While some phytochromes like *Af*Agp1 or *Xcc*BphP also show the interaction between Trp174 and Arg205 (*Is*PadC numbering), others including *Dr*BphP (PDB: 8avw), *At*PhyA (PDB: 8f5z), *At*PhyB (PDB: 7rzw), *Sa*BphP2 PSM (PDB: 6ptx), and *Rp*BphP1 PSM (PDB: 5oy5) lack a tryptophan at this position. Instead, an alternative interaction is observed between the strictly conserved arginine sidechain and the backbone carbonyl of a residue within the GAF β1-β2 turn. Interactions of residues corresponding to *Is*PadC position 175 can be quite diverse as well. The asparagine sidechain observed in *Is*PadC (PDB: 5lly) or *Xcc*BphP (PDB: 6pl0) forms a hydrogen bond to a backbone amide within the “PASDIP” motif. Interestingly, the densities for Asn175 equivalent positions in deposited crystal structures appear to be more defined in Pfr than in Pr structures. In contrast, phytochromes incorporating histidine (*Sa*BphP2 PDB: 6ptx and *Rp*BphP1 PDB: 5oy5) or threonine/serine residues (*Dr*BphP PDB: 4q0J, *Dr*BphP Pfr PDB: 5c5k, and *Af*Agp1 PDB: 5i5l) at the corresponding position lack the previously described interaction due to steric constraints. It should also be noted that the sidechain at position 175 co-defines the environment of the PHY tongue Trp-switch region that is central to the Pr-Pfr transition of the PHY-tongue in phytochromes.^20^

To summarize, the second shell environment and especially the GAF β1-β2 turn co-regulates photochromicity in phytochromes. The mechanism conferred by the β-turn region is the tuning of conformational dynamics in structural elements central to light sensing^50^^,^^51^^,^^52^ thereby affecting important spectral properties such as thermal reversion, which is critical for proper signal integration in many phytochromes,^53^ but also other photoreceptors.^54^ Phytochrome tuning is hypothesized to be initialized by a limited set of interactions with the PASDIP motif. Eventually, these regulatory cues are translated across the whole protein, showcasing the complex allosteric regulation network in phytochromes. These implications should exemplify the potential of the used FSA pipeline which uncovered the discussed β-turn element which is otherwise hidden in the complexity of sequence conservation patterns across phytochrome families. Likely, the unique combination of information from natural and ProteinMPNN sequence conservation will allow the examination of other protein families in future *in silico* studies.

#### ProteinMPNN sequence outputs contain information on position-specific protein properties

In this study, sequence conservation in ProteinMPNN output and natural sequences was employed to infer structural and functional aspects of target proteins in a residue/position-specific manner. In typical applications of ProteinMPNN, amino acid substitutions aiding protein solubility and folding should be inferred, and hence, the generation of sequences encoding highly stable structures is prioritized by the model.^55^ These stability-optimized non-natural protein sequences, consequently, deviate from natural sequences which often trade some stability aspects to enable their functionality that was optimized by evolution.^55^^,^^56^ Hence, comparing natural sequence conservation and ProteinMPNN probabilities provides information concerning the role of sidechains which can lean either in the direction of functional or structural contributions. In a nutshell, residues strictly required for fold stabilization follow more general protein folding principles and can be deduced from the surrounding chemical context, whereas functional residues are missed by ProteinMPNN and rather show up in natural sequence conservation. Depending on the scientific question the FSA workflow is also feasible using LigandMPNN.^36^ However, since cofactor binding is a function of phytochromes the structural classification of ligand-interacting residues in this case appears less intuitive (Figure S6). Especially considering that the class 2 definition as “structural” is intended to reflect protein stability as such and that many phytochromes can be produced as apoproteins, we consider the functional annotation (using ProteinMPNN) to be more generally relevant for ligand binding residues. However, we emphasize that the comparison of results obtained from the two networks can lead to additional interesting insights, especially considering the possibility to run LigandMPNN using input structures corresponding to different functional states (e.g., Pr vs. Pfr in the context of phytochromes). While such comparisons go beyond the scope of this manuscript, they would need a careful analysis of the influence of the neural network weights and/or influence of altered input structure geometries.

Interestingly, already the FSA pipeline based on ProteinMPNN showed its sensitivity to minor variations in the local backbone geometry of input models. The slightly different backbone arrangement for Arg^lasso^ in *Is*PadC and *Af*Agp1 resulted in deviating ProteinMPNN sequence conservation, leading to altered assignments of equivalent positions (see Figure 4B). Another deviating residue, Trp^W(G/A)G^, might be caused by a different tongue conformation in *Af*Agp1 (PDB: 5i5l, 6r26) relative to *Is*PadC, potentially due to coordination of Arg192, Trp^W(G/A)G^, and Pro449. The resulting hydrophobic environment of Trp^W(G/A)G^ was recognized by ProteinMPNN and resulted in diverging class assignments of this position in *Af*Agp1 versus *Is*PadC. Hence, subtle differences in backbone geometries resulting from altered local chemical environments can be identified by the neural network and might allow the identification of tuning residues in allosterically regulated proteins in general. However, careful analysis of such regions would be required, as such results may be influenced by flawed crystallographic data or model inaccuracies of predictions.

The structure-based approach of the FSA pipeline is bolstered by the availability of numerous high-resolution crystal datasets and high-confidence Alphafold2 predictions, which align well with experimental data. In addition, phytochromes deposited in the PDB exhibit a striking structural similarity in their PAS-GAF-PHY arrangement, despite their low sequence similarity. These factors contributed strongly to the extraction of meaningful annotations. This reliance on high-quality crystallographic data or structure prediction models that capture the functional state of interest poses a potential limitation of the FSA pipeline. This is especially true in parts of structural models with low confidence due to increased dynamics or poor sequence coverage in multiple sequence alignments during model generation. The multistate approach partly addresses such uncertainties, as discussed in the original ProteinMPNN publication.^10^ As the neural network considers all models provided in parallel, model imperfection introduced by dynamics or the structure refinement process *et cetera* are averaged out. Another limitation of the workflow is the grouping of amino acids according to a one-dimensional chemical property, as this constitutes an oversimplification, especially for histidine or aromatic residues. While simplifying data analysis, this could be further refined in the future.

A defining advantage of the FSA pipeline is the independent representation of a functional and a structural class of amino acids. One other published study also implemented a representation of structural stability using a vastly different metric, namely Rosetta scores.^5^ However, flagging patterns for the same target protein e.g., *Is*PadC vary substantially between the two approaches, with no easy way to determine which tool represents “the ground truth” more reliably. Overall, of the 17 literature described functional residues, the FSA pipeline captures 17 residues (13 functional/4 structural) and 16 residues (11 functional, 5 structural) in *Is*PadC and *Af*Agp1, respectively. In comparison, the tool released by Cagiada et al.^5^ identified 12 out of 17 residues from the list in Figure 4B and assigns 8 as “functional” and 4 as “structural” (Figure S11). Thus, the fuzziness regarding the delineation of functional or structural contributions is noted in both approaches. While in the case of FSA this can be partly attributed to aromatic residues which are treated in a simplified manner as outlined previously, these issues in both pipelines are a reminder that many residues likely contribute to both roles, functional and structural.

In our test system, the FSA pipeline seems to specifically outcompete the Cagiada tool in the identification of rheostat^44^ and tuning positions in loop/turn regions (Figure S11). For example, positions M190, H193, and the newly described sites within the β-turn (residues 170–175) were not flagged by the energy-based approach. This might stem from a bias in this method toward not flagging polar residues in partly surface exposed loops due to their apparent energetic favorability. Overall, it seems that both the FSA pipeline and the Cagiada tool can empower each other and that the independent evaluation of a protein of interest with both approaches identifies a core set of functionally and structurally relevant positions.

A peculiarity of the FSA approach in the present study was the identification of a third subcategory, “adaptable”, alongside the structural and functional classes. In these positions, amino acid conservation in the ProteinMPNN output is observed in contrast to high diversity in natural sequences. We attribute this to an anchoring effect as the geometry of the input structure at these special positions strongly favors a minimum subset of amino acids, characteristically the same as in the input structure. Typically, the sidechains conserved by ProteinMPNN are glycines, prolines, and the secondary structure-capping residues serine and threonine which are strongly overrepresented in this category (Figure S6). Our reasoning for labeling this class “adaptable” follows our initial hypothesis that nature might have selected against a stable/static structure in these regions. Yet, it cannot be ruled out that this category rather represents artifacts of the input structure, potentially even originating from the structure refinement/generation process. In addition, it should be noted that the filtering thresholds used in the FSA pipeline primarily affect the absolute number of “adaptable” residues. In several instances these are edge-cases where the natural enrichment is close to statistical significance and a potential “structural” assignment. Therefore, also the diversity of sequences being compared, the size of the input alignment, and the quality of its curation play a central role for the FSA pipeline and the empirical thresholds being applied. However, assessing all these parameters and their implications especially on the “adaptable” category would require substantial further experiments that are beyond the scope of the present study.

Another peculiar behavior of the FSA pipeline unfolds when analyzing *Is*PadC position 172 highlighted in Figure 2B. This position is flagged as functional since the natural sequences show significant enrichment of the negatively charged chemical group whereas in the ProteinMPNN sequences no significant conservation is observed. This is the defined criteria to flag this position as functional. However, *Is*PadC harbors a proline at this position which deviates from most related phytochromes which prevalently show aspartate or glutamate. Therefore, the position is flagged because of the amino acid conservation in other related phytochromes. In this case, this position might represent an evolutionary playing ground as the rare insertion of proline might have removed interactions which might otherwise affect spectral and/or other functional properties. This might also explain why the P172A variant did not strongly influence the phytochrome properties tested in this study. Several functional positions follow the same behavior where the flagging is triggered by the alignment and *Is*PadC shows a less prevalent residue. However, these observations are still valuable in highlighting important residues where *Is*PadC deviates from other homologs.

In the wider context, large language models (LLMs) seem like another viable option to assign functional and structural aspects in proteins. In fact, the conservation of important positions in computationally redesigned sequences was already observed in prior studies using LLMs like ESM-1b^57^ and the ProGen pipeline.^7^ In the latter, novel generated sequences exhibit preferential conservation of buried versus non-buried positions.^7^ However, both ligand-interacting and core-packing residues were captured that influence functional and structural aspects, respectively. The ProGen pipeline was not designed to delineate these categories, yet this behavior might indicate potential limitations of LLMs for this purpose. As specifically shown for ESM 1b, LLMs seem to encode and recapitulate the alignment information of whole protein families. Thereby, protein-specific functional “slang” is inadvertently co-encoded, which might blur the differentiation between functional and structural residues. Therefore, our 3D coordinates-based approach, though having other limitations, might have an advantage in being less promiscuous.

### Conclusion and significance

In summary, we are confident that the presented FSA pipeline can be valuable in uncovering less obvious functional hotspots in a range of interesting target proteins. This area of research is of general interest as it might allow *in-silico* characterization of disease-causing protein variants, tuning of enzyme activities for industrial applications, and many other protein functionalization aspects. In the future, a more detailed characterization of residues falling into the classes of “structural” and “adaptable” could also further enlighten our appreciation of central aspects of the structure-function relationship and their link to intrinsic conformational dynamics in proteins. To conclude, our workflow might be an additional step toward an improved understanding of the intricate inner workings of life itself.

## Resource availability

### Lead contact

Further information and requests for resources and reagents should be directed to and will be fulfilled by the lead contact, Andreas Winkler (andreas.winkler@tugraz.at).

### Materials availability

Plasmids for the expression of the protein variants described herein are available upon request.

### Data and code availability

• All data needed to evaluate the conclusions of the paper are present in the paper and/or the Supporting Information file. All data used in the analyses are publicly available as of the date of publication in the public repository of the Graz University of Technology under https://doi.org/10.3217/432fm-rms13.
• The original code generated within this study and example input files are publicly available as of the date of publication at https://gitlab.tugraz.at/bioc/fsa.
• Any additional information required to reanalyze the data reported in this paper is available from the lead contact upon request.

## Acknowledgments

We would like to thank Heikki Takala for providing the raw data of the *Af*Agp1 spectrum. In addition, we want to thank Aleksandar Bijelic for rigorous proof-reading of the manuscript. M.G.T. and O.M.E. are supported by the 10.13039/501100002428Austrian Science Fund (FWF) grant https://doi.org/10.55776/DOC130 and M.G.T. additionally by the Styrian Government (Amt der steiermärkischen Landesregierung, Zukunftsfonds, doc.fund program). O.M.E. and M.G.T. were trained within the framework of the PhD program Biomolecular Structures and Interactions (BioMolStruct). G.O. was supported by funding from the European Research Council through a Starting Grant (HelixMold 802217). This research was funded in whole, or in part, by the Austrian Science Fund (FWF) (https://doi.org/10.55776/P32022 to A.W. and https://doi.org/10.55776/P30826 to G.O.). For open access purposes, the authors have applied a CC BY public copyright license to any author accepted manuscript version arising from this submission.

## Author contributions

Conceptualization computational part, M.G.T.; conceptualization of in vitro experiments, O.M.E.; methodology, M.G.T. and O.M.E.; validation M.G.T. and O.M.E.; formal analysis M.G.T. and O.M.E.; investigation, M.G.T., O.M.E., and S.M.; writing-original draft, O.M.E. and A.W.; writing-review and editing, O.M.E. and A.W.; visualization, O.M.E.; supervision, A.W. and G.O.; funding acquisition, A.W. and G.O.

## Declaration of interests

The authors declare no competing interests.

## Declaration of generative AI and AI-assisted technologies in the writing process

During the preparation of this work, the author(s) used ChatGPT-3.5 in order to check spelling, grammar, and conciseness and improve readability of the manuscript. After using this tool/service, the author(s) reviewed and edited the content as needed and take(s) full responsibility for the content of the published article.

## STAR★Methods

### Key resources table

**Table:** 

REAGENT or RESOURCE	SOURCE	IDENTIFIER
**Bacterial and virus strains**		

BL21 (DE3)	Thermo scientific™	Catalog number EC0114
BL21 (DE3) pT7-ho1	Gourinchas et al.^39^	N/A

**Chemicals, peptides, and recombinant proteins**		

*Is*PadC PSM	This paper	N/A
*Is*PadC PSM P172A	This paper	N/A
*Is*PadC PSM E173A	This paper	N/A
*Is*PadC PSM W174G	This paper	N/A
*Is*PadC PSM N175A	This paper	N/A
*Is*PadC PSM R177Q	This paper	N/A
*Is*PadC PSMM190Y F259M	This paper	N/A

**Deposited data**		

Raw data for individual figures; TU Graz Repository	This paper	https://doi.org/10.3217/432fm-rms13
FSA algorithm	This paper	https://gitlab.tugraz.at/bioc/fsa

**Oligonucleotides**		

Primers to generate point mutations see Table S1	This paper	N/A

**Recombinant DNA**		

pETM11 *Is*PadC PSM (and variants)	This paper	N/A
pT7-ho1	Tarutina et al.^58^	N/A

**Software and algorithms**		

Alphafold 2 Colab	Mirdita et al.^14^	https://colab.research.google.com/github/sokrypton/ColabFold/blob/main/AlphaFold2.ipynb
Chai	Boitreaud et al.^37^	https://lab.chaidiscovery.com/auth/login?callbackUrl=https%3A%2F%2Flab.chaidiscovery.com%2Fdashboard
ProteinMPNN	Dauparas et al.^10^	https://github.com/dauparas/ProteinMPNN; model_name v_48_020
Origin	OriginLab	https://www.originlab.com/
CorelDraw	Alludo	https://www.coreldraw.com
Psi-blast CLI tool	Altschul et al.^59^	https://packages.debian.org/
Hexicon 2	Lindner et al.^60^	http://hx2.mpimf-heidelberg.mpg.de
T-COFFEE Multiple Alignment Sequence Server	Notredam et al.^61^	https://tcoffee.org/
SWISS-MODEL Webserver	Arnold et al.^62^	https://swissmodel.expasy.org/interactive
Jalview	Waterhouse et al.^63^	www.jalview.org

### Experimental model and study participant details

#### Microbe strains

*E. coli* BL21 (DE3); *E. coli* BL21 (DE3) pT7-ho1 as described in Gourinchas et al. 2017.^39^

### Method details

#### Phylogenetic analysis and FSA workflow

For the natural sequence pipeline, sequence retrieval and multiple sequence alignments were performed as described previously.^32^ In brief, an iterative PSI-blast^59^ search was performed for retrieving sequences from the NCBI website. Alignments were performed with T-COFFEE Multiple Alignment Sequence Server^61^ including structural information with advanced settings (specifics see^32^) followed by manual curation in Jalview.^63^

For the non-natural sequence pipeline, target sequences (*Is*PadC PSM, *Af*Agp1 PSM) were provided to the Alphafold2 Colab^14^ and predicted as dimers with standard settings. When using crystallographic data, gaps in the data was filled by creating models in the SWISS-MODEL^62^ webserver. Highest ranking relaxed output structures of Alphafold2 and models created from solved crystal structures (*Is*PadC: 5llw, 5lly; *Af*Agp1: 5I5l, 6r26) were combined into one PDB file containing in total 3 dimeric models per target. This master file was provided to a local ProteinMPNN installation and run as a “multistate design” approach. A total of 1000 *de novo* sequences were generated for each target which is followed by functional classification automated in a jupyter python notebook, available at https://gitlab.tugraz.at/bioc/fsa.

For the analysis, the input MSAs generated for both natural and *de novo* sequences need to be precomputed in the standard psiblast PSSM format. This computation was performed and tested with the Psi-blast^59^ CLI tool as provided in the blast v2.5.0 Debian package. The FSA script loads the two PSSMs (alignment- and ProteinMPNN-derived), analyses them separately, to identify significant patterns and encodes the information contained therein. Residues are clustered according to their physico-chemical similarity, resulting in the following classes: acidic, basic, hydrophilic, hydrophobic, glycine and proline. Important positions are identified by comparing these groups distributions to the full protein baseline, statistical significance is defined as p-scores <0.01 on a non-parametric Mann-Whitney U test. These positions are further filtered by considering the PSSM-calculated information score to scale the differential distribution Frobenius norm and discarding a portion of positions in the sequence which is definable by a threshold in the python script. The exact threshold depends on the depth and variability of the evolutionary sequence alignment and can be optimized using known functional or structural residues. The encoded PSSMs are then compared to classify the residues flagged as significant in either of them. Each position is classified as “functional” if it is significant in the natural sequence alignment PSSM, but not in the ProteinMPNN output PSSM, as “adaptable” if it’s the other way around and as “structural” if it is significant in both. The results are then plotted in a color-coded heatmap graph with functional residues highlighted in pink, structural residues in metallic blue and adaptable residues in yellow.

#### Cloning, expression, and purification

The wildtype sequence of *Idiomarina* sp. A28L PadC (WP_007419415) in the pETM11 vector system first described in Gourinchas et al.^39^ was truncated and point mutations introduced according to a protocol described by Liu and Naismith.^64^ A list of primers can be found in Table S1. We generated multiple PAS-GAF-PHY variants including an N-terminal His_6_ tag and omitting the coiled-coil linker sequence and the effector domain. The resulting constructs extending to amino acid Leu500 are termed *Is*PadC PSM herein.

Expression of protein variants was conducted in the strain BL21 (DE3) pT7-ho1 as published in^39^ which contains a helper plasmid encoding a heme oxygenase (HO-1) from Synechocystis sp. PCC6803 for efficient biliverdin-IXα production. Growing conditions involve a non-actinic dim green light environment and LB media (“Lennox”, Roth; 10 g/L Trypton, 5 g/L Yeast extract, 5 g/L NaCl) supplemented with kanamycin (34 μg/L), 8.5 mM MgCl_2_ and 0.3% glucose. Cultures are grown at 37°C at 130 rpm until reaching an optical density of 0.5 followed by addition of δ-aminolevulinic acid (10 mg/L) and cooling to 18°C for 30 min. Upon addition of isopropyl-β-D-thiogalactopyranoside (IPTG, 0.25mM), cultures are incubated at 130 rpm and 18°C for 14–18 h.

Bacterial pellets were harvested by centrifugation at 5,000 RCF at 8°C. For cell lysis the bacteria were resuspended in lysis buffer (50 mM HEPES pH 7.0, 500 mM NaCl, 2 mM MgCl_2_, 10 mM Imidazol) containing lysozyme (100 μg/mL) and DNase (100 μg/mL). Cells were disrupted by sonication (4 × 5min, 50W, Labsonic LU, 0.7s duty cycle, ice water cooling) and bacterial debris removed by centrifugation (39,000 RCF, 4°C, 1 h). The Holoprotein was purified using Ni^2+^-sepharose matrix in a gravity flow setup. In short, cleared cell lysate was loaded onto the column, followed by 10 column volumes of wash buffer (50 mM HEPES pH 7.0, 500 mM NaCl, 2 mM MgCl_2_, 50 mM imidazol). Addition of elution buffer (50 mM HEPES pH 7, 500 mM NaCl, 2 mM MgCl_2_, 250 mM imidazol) then removes the protein of interest from the matrix. Elution fractions were concentrated using centrifugal filters (Amicon MW cut-off 30,000 Da, 4000 RCF) and further purified by size exclusion chromatography on a Superdex 200 Increase 10/300GL column equilibrated in size exclusion buffer (10 mM HEPES pH 7.0, 500 mM NaCl, 2 mM MgCl_2_). Monodisperse peak fractions were concentrated by centrifugal filtration as described above and the protein was flash frozen in liquid nitrogen and stored at −80°C for further characterization.

#### Ultraviolet-visible (UV-vis) absorption spectroscopy

UV-vis spectra were collected on a Specord 200plus spectrophotometer with 1 nm spacing at a scan rate of 200 nm/s and an integration time of 5 ms. Protein samples were measured in a quartz cuvettes diluted to 2 μM in 500 μL of size exclusion buffer (see above). Pr samples are measured using dark adapted samples, Pfr-enriched PSS samples were generated by illumination with red light (660 nm, 20 mW/cm^2^, Thorlabs) for 1 min before measurement.

Pr recovery rates, also termed thermal reversion, were recorded by illuminating samples for 1 min with red light before following absorbance at two wavelengths (705 nm and 750 nm) in given time intervals. These wavelengths were chosen as a compromise for slightly deviating absorption spectra of *Is*PadC PSM variants. Time intervals are chosen according to the thermal reversion characteristics in order to have comparable amounts of datapoints for the non-linear fit, and only marginal actinic effects due to the measuring light.

#### Fluorescence measurements

An RF-6000 spectrofluorimeter (Shimadzu) with a 150 W xenon arc lamp was used for measuring excitation and emission spectra of protein variants adjusted to 2 μM in size exclusion buffer. Dark adapted samples equilibrated at room temperature were measured with a slit width of 5 nm for the excitation and emission experiments, a scanning speed of 200 nm/min, sensitivity “high”, and a data interval of 1 nm. Emission spectra were determined after 670 nm excitation whereas excitation spectra were recorded at a constant emission wavelength of 740 nm.

#### Hydrogen deuterium exchange MS

A detailed description of the workflow is provided in Fuchs and Winkler.^65^ Briefly, protein variants were adjusted to 40 μM protein concentration with size exclusion buffer (see above) and 5 μL were aliquoted in 1.5 mL reaction tubes followed by flash freezing with liquid nitrogen. These samples were prepared in triplicates for each of the 5 time points to be measured. General sample treatment was performed as follows; samples were quickly thawed before being equilibrated for 1 min at 20°C. The dark series samples were equilibrated under non-actinic green light conditions whereas light samples were continuously illuminated with red light (2 mW/cm^2^) during equilibration and the subsequent labeling reaction. Then 95 μL labeling buffer (10mM HEPES pD 7.0, 150 mM NaCl, 10 mM MgCl_2_) prepared in D_2_O were added. After time points of 10 s, 45 s, 3 min, 15 min, and 60 min 16 μL of the labeling mix were pipetted into a tube containing 16 μL of quenching buffer (200 mM ammonium formate, pH 2.6) followed by immediate flash freezing in liquid nitrogen. For measurement in the LC-MS setup, quenched samples were thawed by addition of 80 μL quenching buffer and injection of 100 μL into a cooled HPLC system as described previously in.^66^

Prior to HPLC separation, the injected samples were subjected to online protease cleavage on an immobilized pepsin column (BEH Enzymate, Waters) at 10°C with a flow rate of 0.3 mL/min. Peptide fragments were desalted on a C18 trap column (Shim-pack GISS-HP(G), Shimadzu). Separation of peptides eventually occurred on a C18 reversed-phase column (Shim-pack Arata Peptide, Shimadzu) utilizing a 4.25 min acetonitrile gradient (from 10% to 45%) with a constant amount of 0.6% (v/v) formic acid. Eluting protein products were measured on an Impact II ESI-Q-TOF (Bruker) mass spectrometer and data files were exported to mzxml format for further analysis. We quantitated deuterium incorporation using the Hexicon 2 software package.^60^ By injection of blanks, sample carry over between runs was confirmed to be less than 10% and back-exchange was estimated to be around 30% based on the characteristics of fully exchanging peptides. An overview of HDX-MS statistics is depicted in Table S2.

### Quantification and statistical analysis

#### Hydrogen deuterium exchange data

Relative deuterium uptake values in Figure S9 are shown as the mean of three independent measurements and error bars correspond to the sample standard deviation as generated using the software package Hexicon 2.^60^ This information can also be found in the figure legend of Figure S9. Additional details of HDX measurements are provided in Table S2.

## Supplemental information

Document S1. Figures S1–S11 and Tables S1 and S2

Document S2. Article plus supplemental information
