# Supplementary material for: Integrating protein sequence design and evolutionary sequence conservation to uncover spectral tuning sites in red-light photoreceptors
Source: Structure. 2025 Nov 6;33(11):1916–1929.e3. doi: 10.1016/j.str.2025.07.018 (PMC12617373; doi:10.1016/j.str.2025.07.018)
Supplement: Document S1. Figures S1–S11 and Tables S1 and S2 [file mmc1.pdf]

Structure, Volume *33*

Supplemental Information

Integrating protein sequence design and evolutionary sequence conservation to uncover spectral tuning sites in red-light photoreceptors

Oliver Maximilian Eder, Massimo Gregorio Totaro, Stefan Minnich, Gustav Oberdorfer, and Andreas Winkler

Integrating protein sequence design and evolutionary sequence conservation to uncover spectral tuning sites in red-light photoreceptors

Oliver Maximilian Eder[1], Massimo Gregorio Totaro[1], Stefan Michael Minnich[1], Gustav Oberdorfer[1,2], and Andreas Winkler[1,2]

Affiliations
[1] Graz University of Technology, Graz, Austria
[2] BioTechMed Graz, Graz, Austria

Supporting information

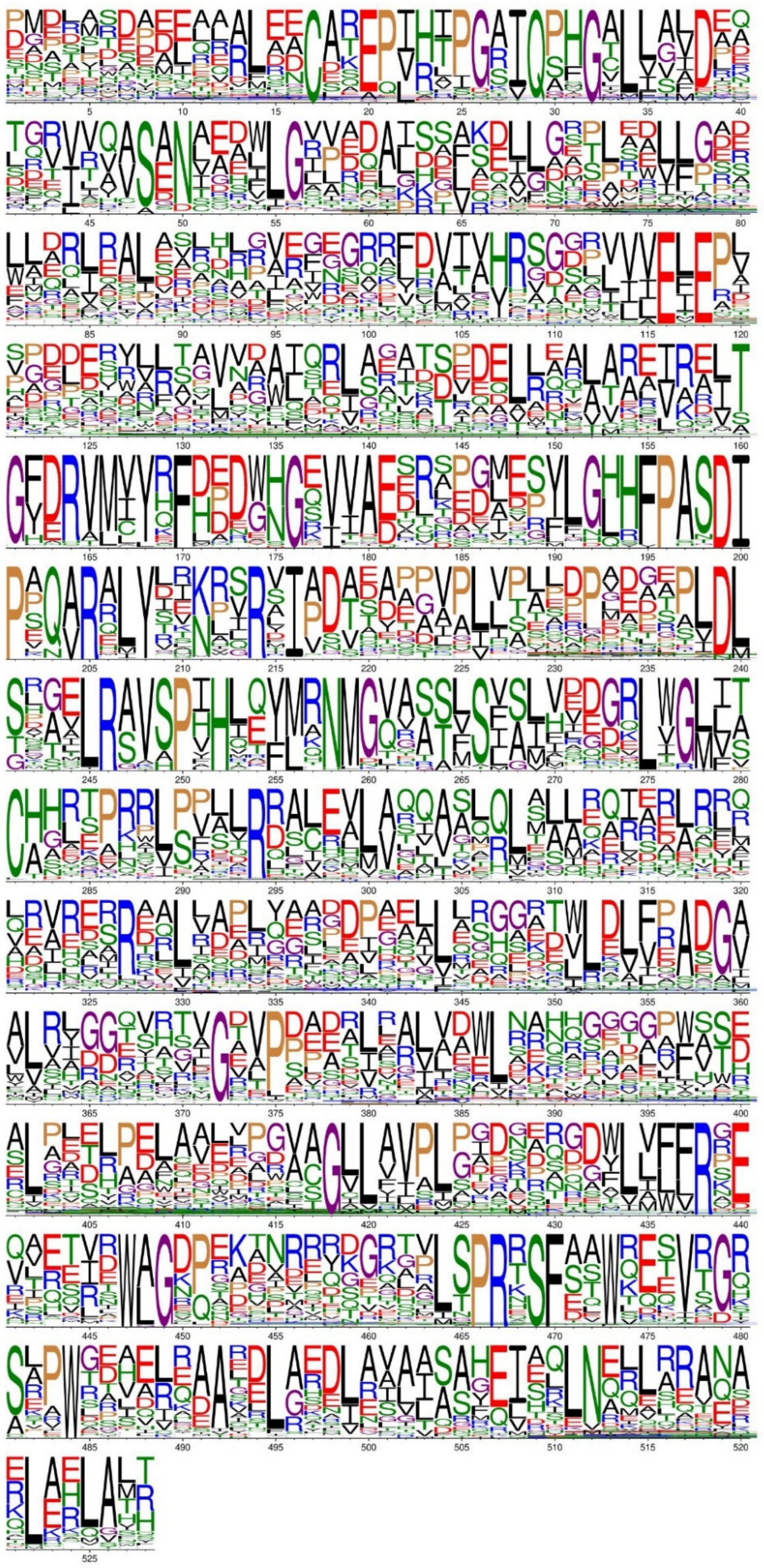

Figure S1: **Weblogo depiction of natural sequences of the PadC protein family,** related to Figure 2A+B and STAR methods. Natural sequences were retrieved from repositories and aligned as outlined in the Materials and Methods section. Weblogo colors depict the chemical classes of amino acids as follows: hydrophobic residues (black), negatively charged residues (cherry red), positively charged residues (dark blue), polar residues (green), glycine (purple), proline (brown).

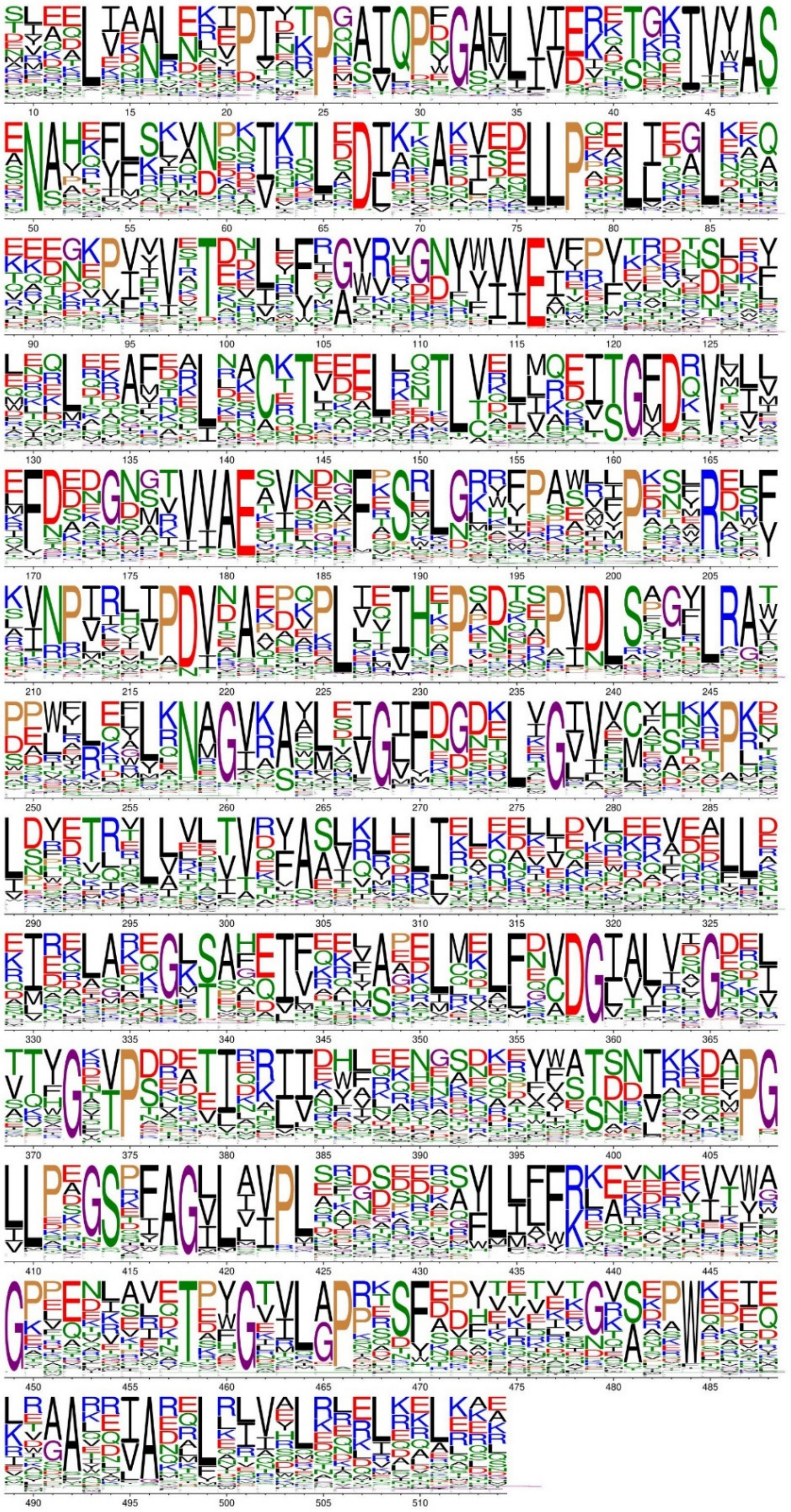

Figure S2: **Weblogo depiction of ProteinMPNN sequences generated with *Is*PadC as input coordinates,** related to Figure 2A+B and STAR methods. 1000 output sequences were generated by ProteinMPNN which should structurally recapitulate *Is*PadC. Weblogo colors depict the chemical classes of amino acids as follows: hydrophobic residues (black), negatively charged residues (cherry red), positively charged residues (dark blue), polar residues (green), glycine (purple), proline (brown).

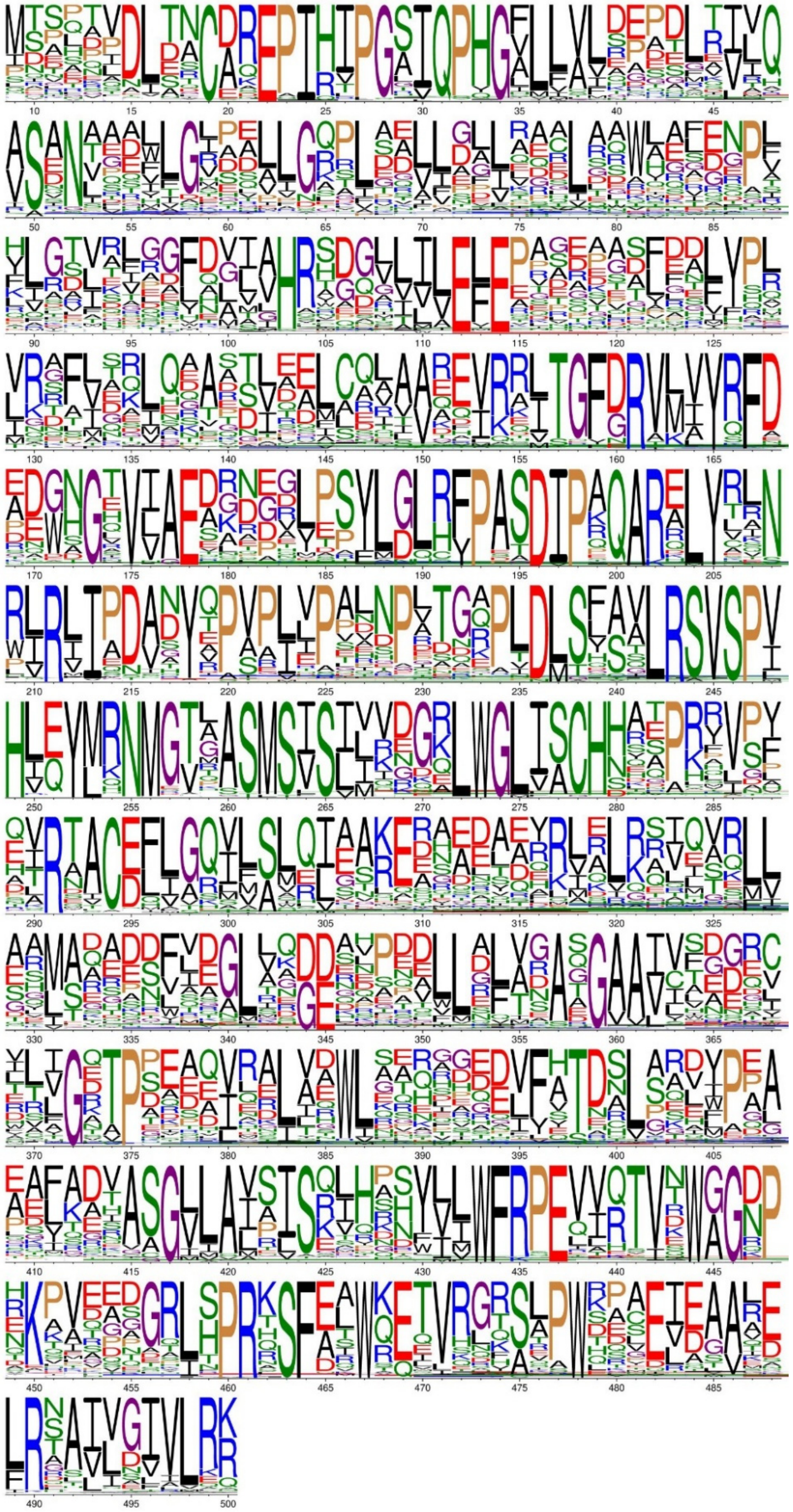

Figure S3: **Weblogo depiction of natural sequences of *Af*Agp1 and homologues,** related to Figure 2A+B and STAR methods. Natural sequences were retrieved from repositories and aligned as outlined in the Materials and Methods section. Weblogo colors depict the chemical classes of amino acids as follows: hydrophobic residues (black), negatively charged residues (cherry red), positively charged residues (dark blue), polar residues (green), glycine (purple), proline (brown).

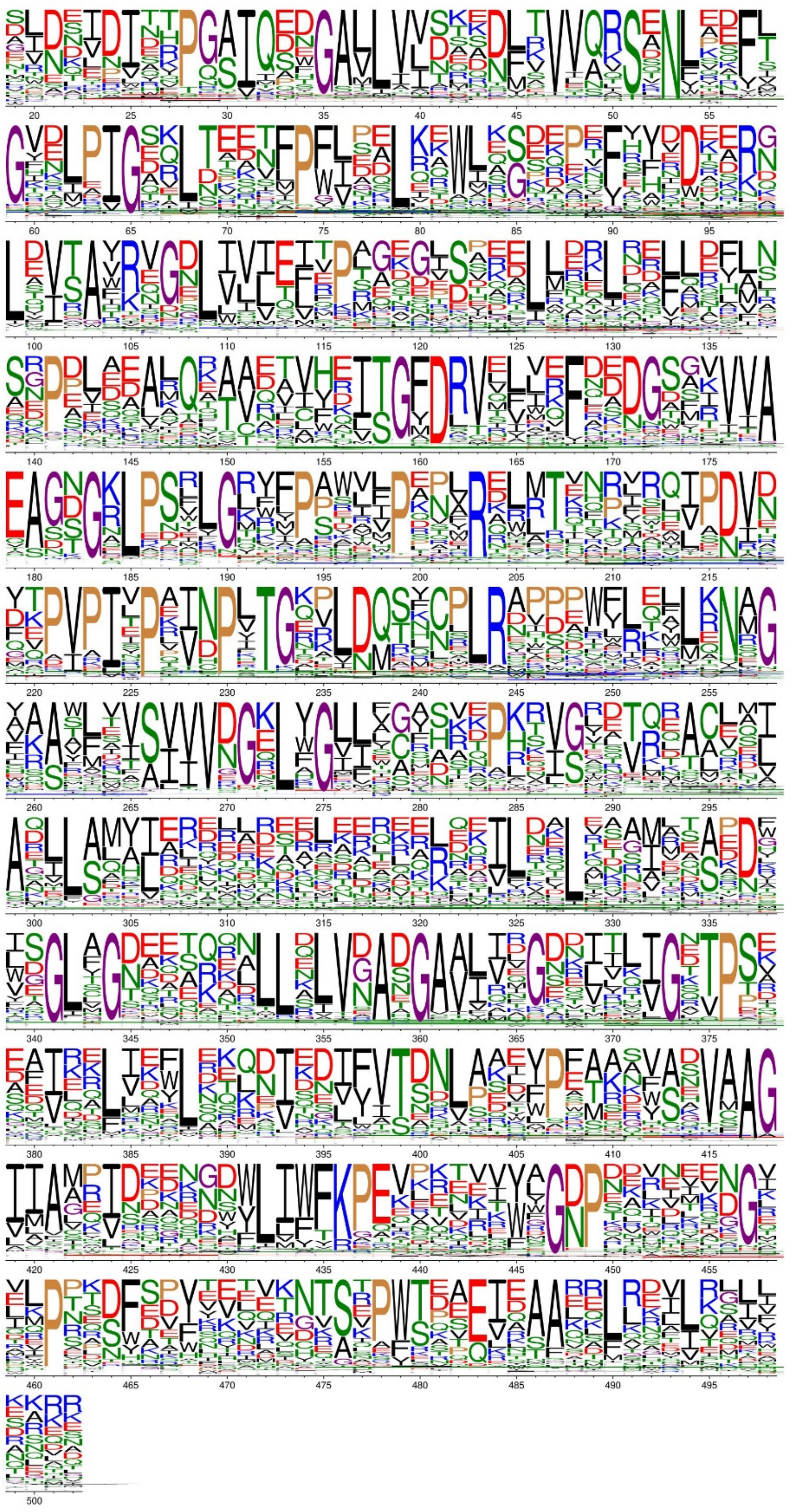

Figure S4: **Weblogo depiction of ProteinMPNN sequences generated with *Af*Agp1 as input coordinates,** related to Figure 2A+B and STAR methods. 1000 output sequences were generated by ProteinMPNN which should structurally recapitulate *Af*Agp1. Weblogo colors depict the chemical classes of amino acids as follows: hydrophobic residues (black), negatively charged residues (cherry red), positively charged residues (dark blue), polar residues (green), glycine (purple), proline (brown).

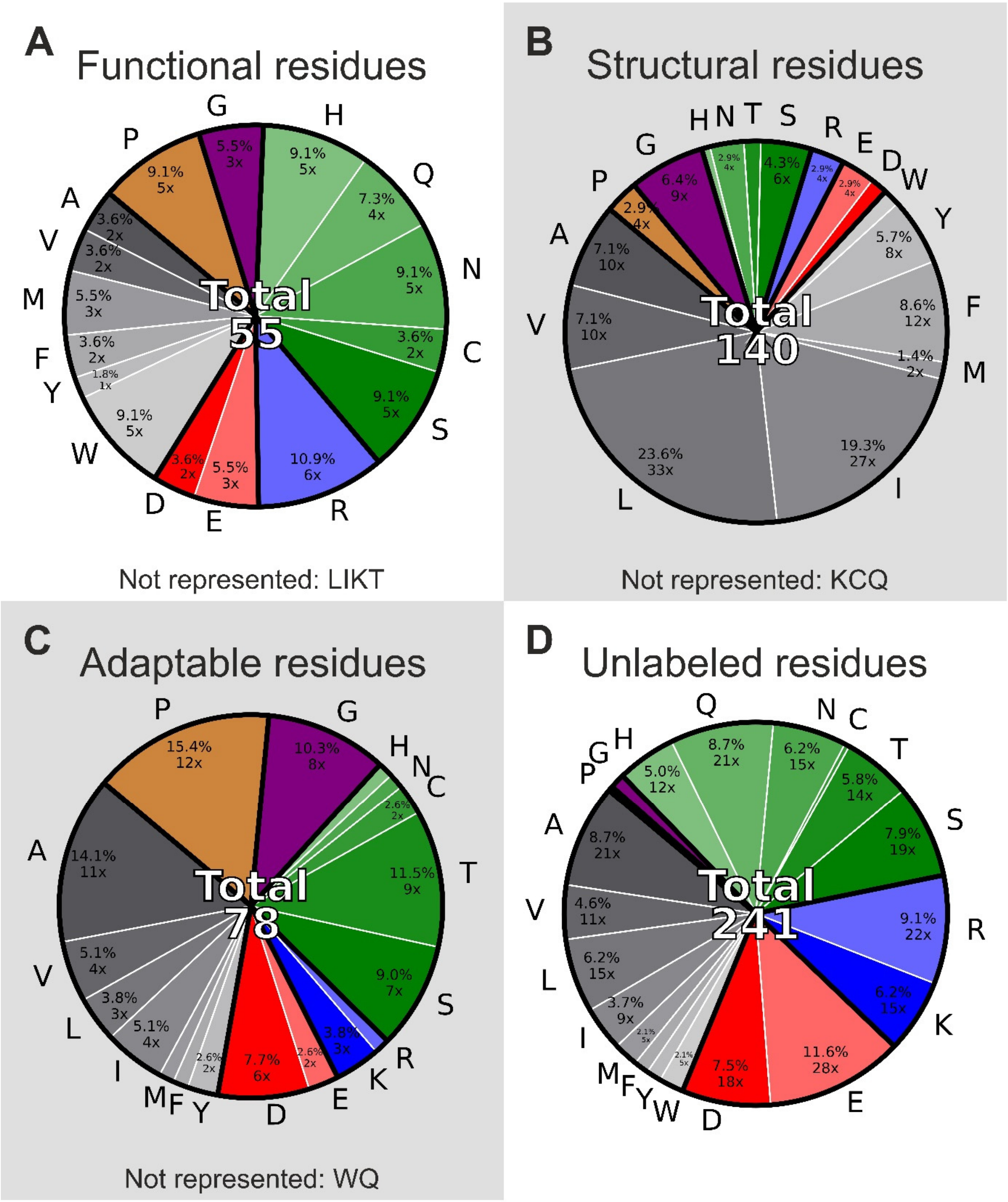

Figure S5: **Statistics of the FSA analysis conducted on *Is*PadC including all classes,** related to Figure 3C+D. A-D) Amino acid composition of the assigned classes for functional, structural residues, adaptability and unflagged residues, respectively. Amino acid frequencies are depicted as percentages of the total number shown in the middle of the pie chart and as absolute numbers (e.g. 5x). Percentage values lower than 2% are not displayed in the figure.

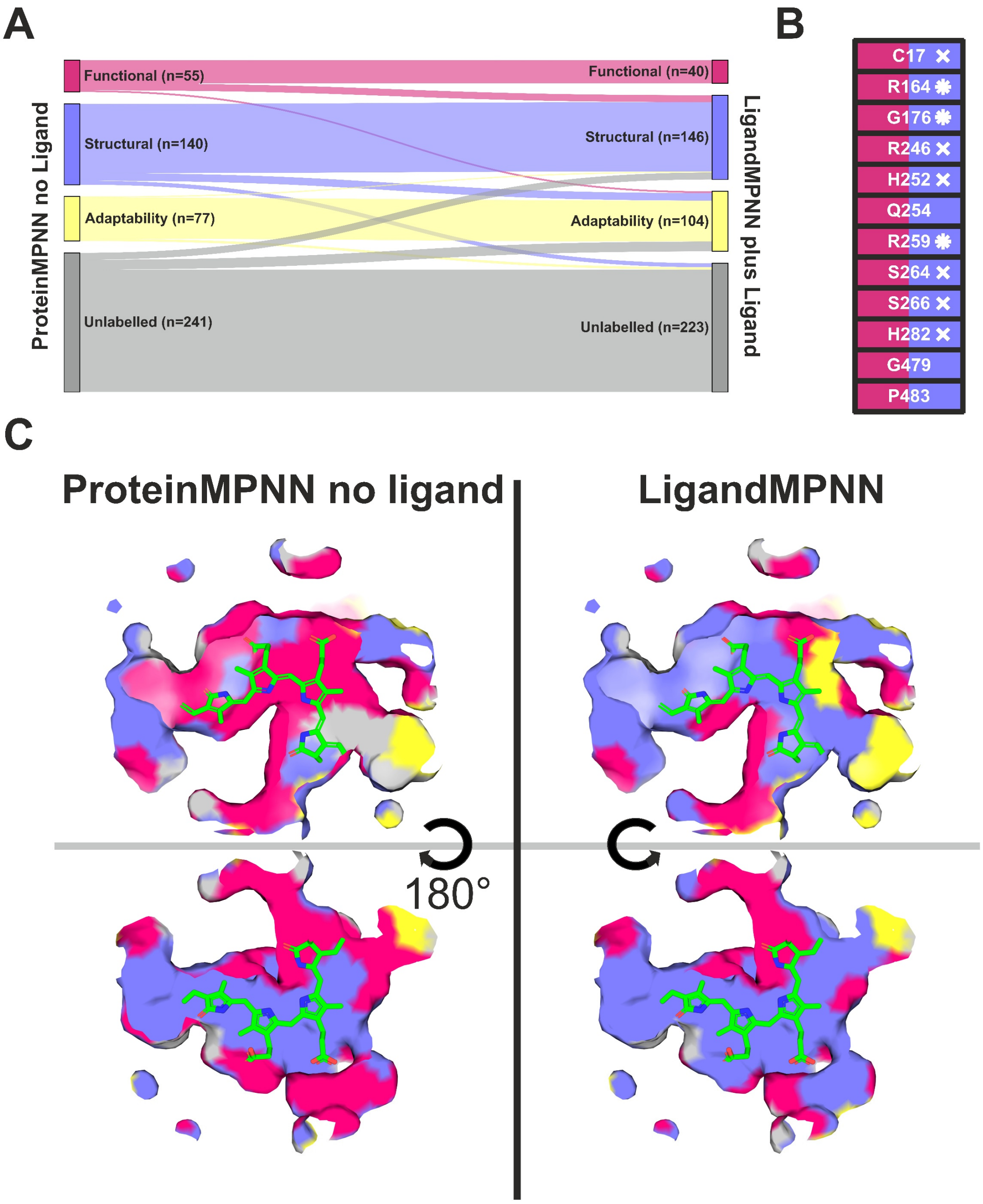

Figure S6: **ProteinMPNN- versus LigandMPNN-based FSA annotation,** related to Figure 4. A) Overall comparison of assigned classes when using different neural networks. In total, 71 residues change their classification due to the usage of LigandMPNN and an input file including the BV cofactors. B) Residues changing their classification from functional to structural (ProteinMPNN versus LigandMPNN). Of the 12 residues changing classification, nine highlighted residues can be directly explained by the incorporation of the ligand in the input model as LigandMPNN generates sequences optimized for binding affinity and structural compatibility with ligands (X – direct interactions; Star – close proximity). Hence, they change classification from functional to structural. C) FSA annotation of the ligand binding cavity of *Is*PadC when using ProteinMPNN versus LigandMPNN. In B), LigandMPNN recognized the importance of the 9 highlighted residues in the ligand binding pocket, the resulting change in annotation from functional (pink) to structural (metallic blue) is visible in the colored ligand binding pocket.

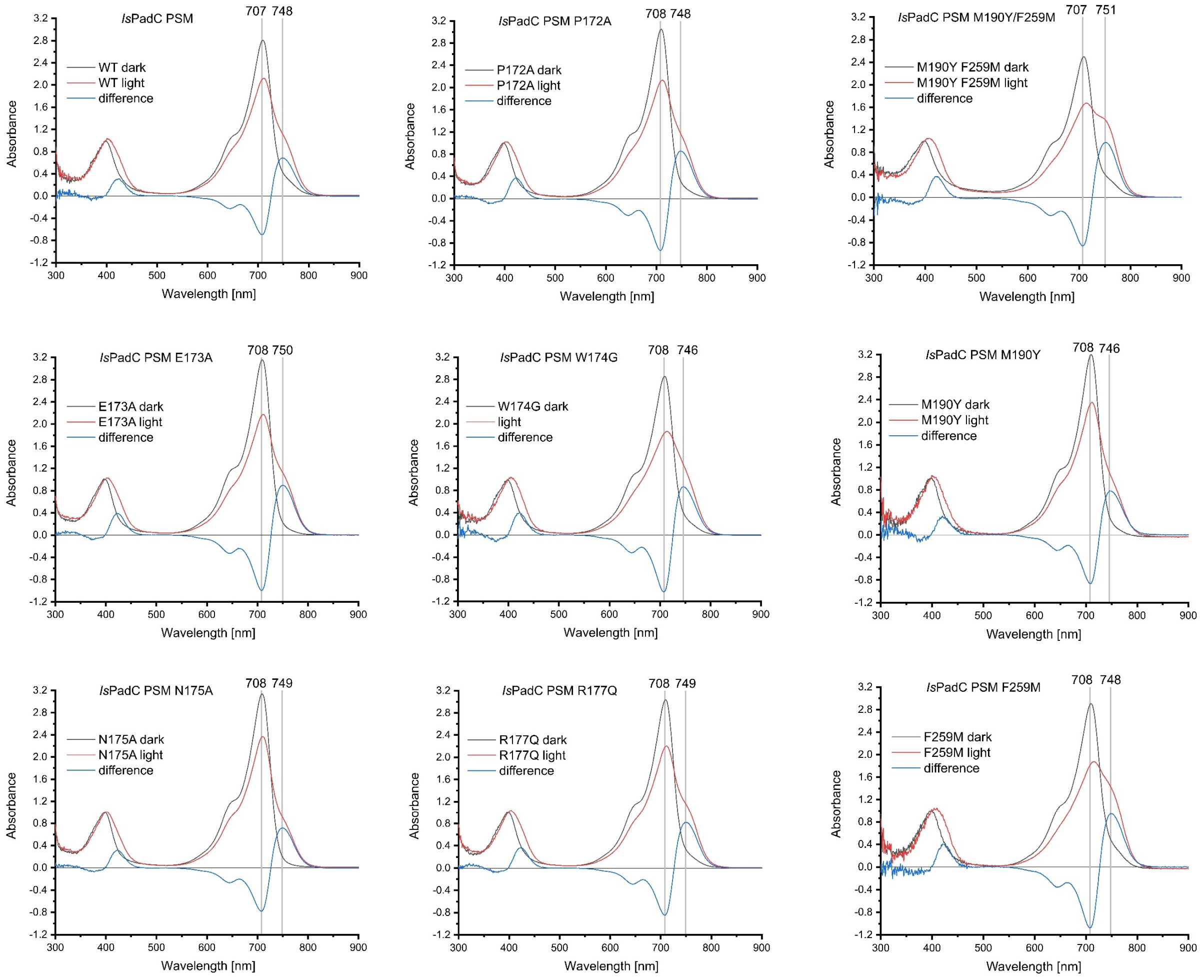

Figure S7**: UV/vis spectra of *Is*PadC PSM variants,** related to Figure 5. A-F) UV/vis absorption spectra of all *Is*PadC variants discussed in this manuscript. In all panels, the dark-adapted state is depicted as black line whereas the red light induced PSS^{660nm} is shown as red line. Difference spectra (light minus dark) are depicted in blue and with grey line the maxima and minima of the difference spectrum is shown. Spectra are scaled to 1 according to their Pr 398 nm Soret band maximum.

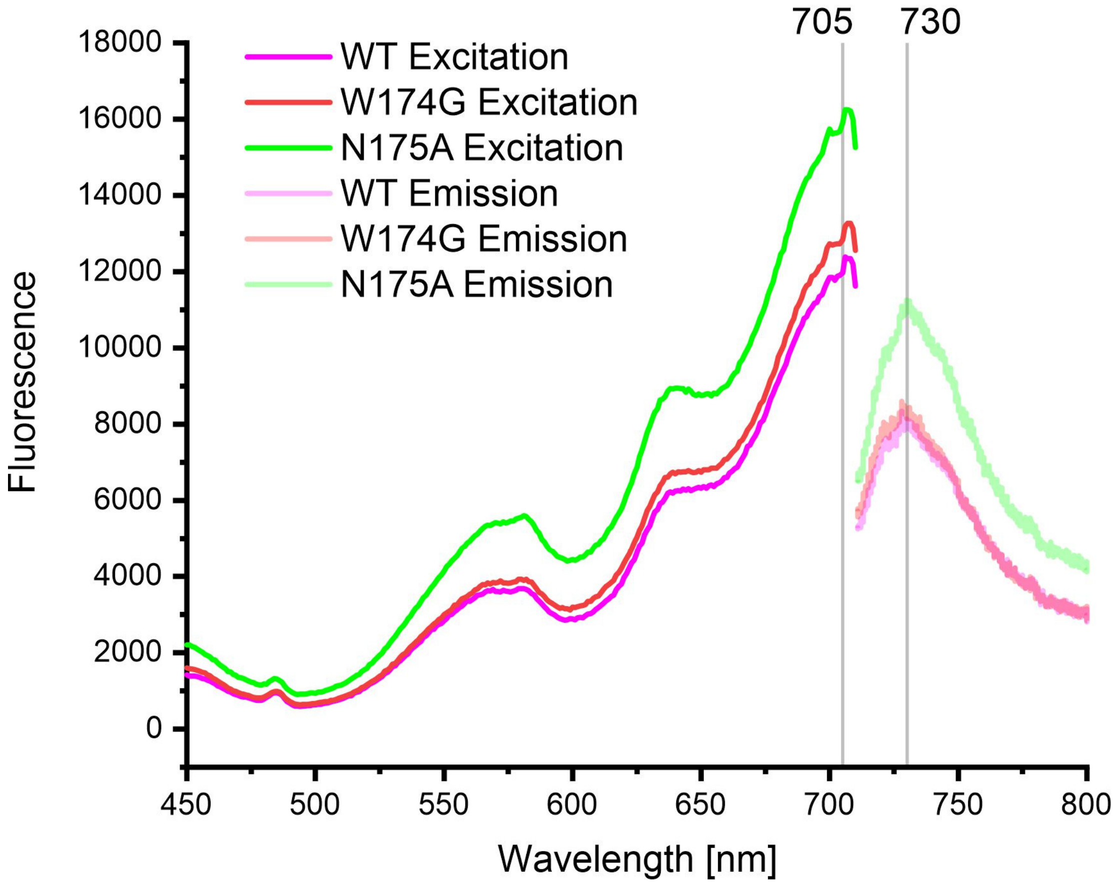

Figure S8: **Fluorescence Emission and Excitation traces of *Is*PadC WT and variants,** related to Figure 5. Fluorescence emission intensity and extinction spectra were recorded for dark adapted samples of *Is*PadC wildtype and variants W174G and N175A. Note that variant W174G shows WT-like emission intensity whereas N175A harbors slightly increased fluorescence emission.

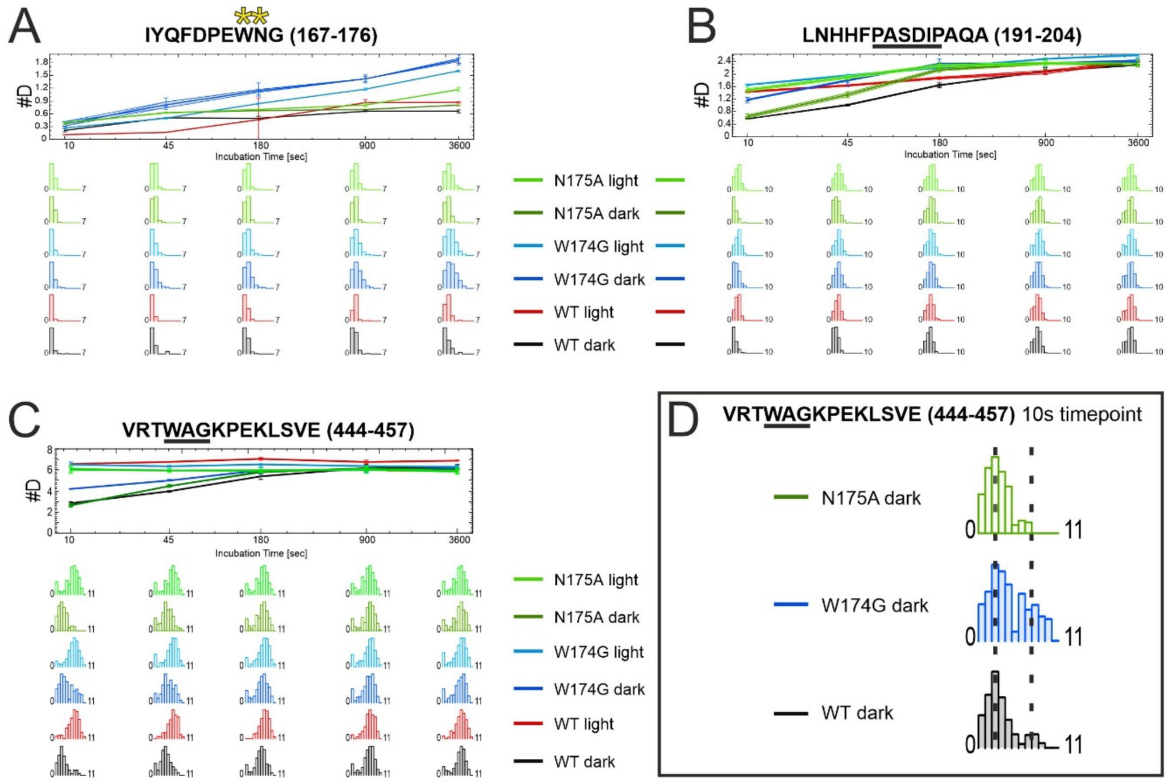

Figure S9: **Detailed hydrogen-deuterium exchange MS results,** related to Figure 6. A-C) Deuterium incorporation kinetics plotted against time for selected key light sensing peptides in the phytochrome *Is*PadC. These peptides are: the β-turn (residues 170-176) which was targeted by mutagenesis (substituted positions are highlighted with yellow asterisks), the PASDIP motif, and the WAG motif of the PHY tongue. Note the change in scaling of the plots, with peptides (A) and (B) showing low deuterium incorporation, whereas peptides in (C) show high overall deuterium incorporation. Relative deuterium uptake values (#D) are shown as the mean of three independent measurements and error bars correspond to the sample standard deviation. Distributions of deuterium-incorporating peptide populations are shown below the time-dependent plots. D) Magnified view of the deuterium incorporation distribution at 10 s in the dark state of a WAG motif-containing peptide. Peaks of the distributions are indicated by black dotted lines.

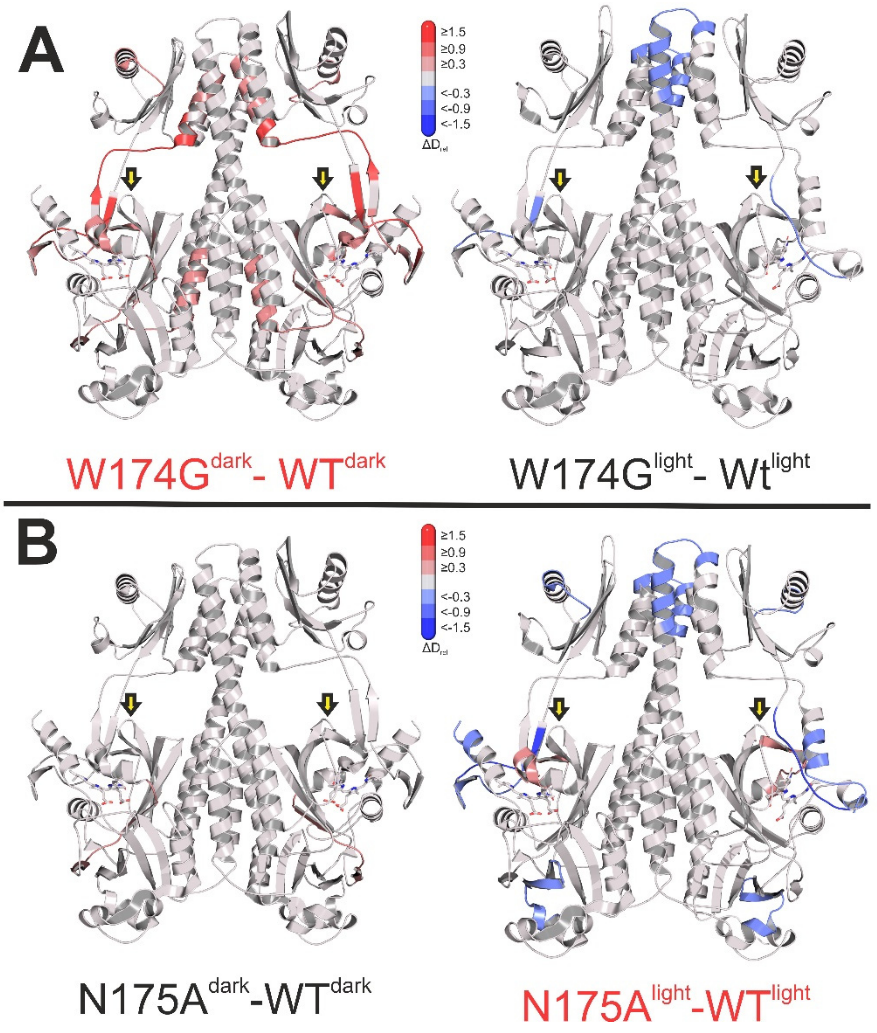

Figure S10: **HDX-MS characterization of *Is*PadC PSM variants in dark and light states,** related to Figure 6. A) Differences in deuterium exchange (ΔD_{rel}) of *Is*PadC PSM W174G minus WT data in the dark (t = 10 s) and with red light illumination (t = 10 s) mapped onto the structure of *Is*PadC. B) Differences in deuterium exchange (ΔD_{rel}) of *Is*PadC PSM N175A minus WT datasets in the dark (t = 3 min) and with red light illumination (t = 3 min) mapped onto the structure of *Is*PadC. Red text indicates the datasets already featured in the main text. The positions targeted by substitutions are highlighted by yellow arrows. Red and blue regions, according to the bar legend, highlight more and less deuterium incorporation in the test sample versus the WT reference, respectively. PDB structures 5llw (dark adapted) and 6et7 (red light-illuminated) were used as models for visualization.

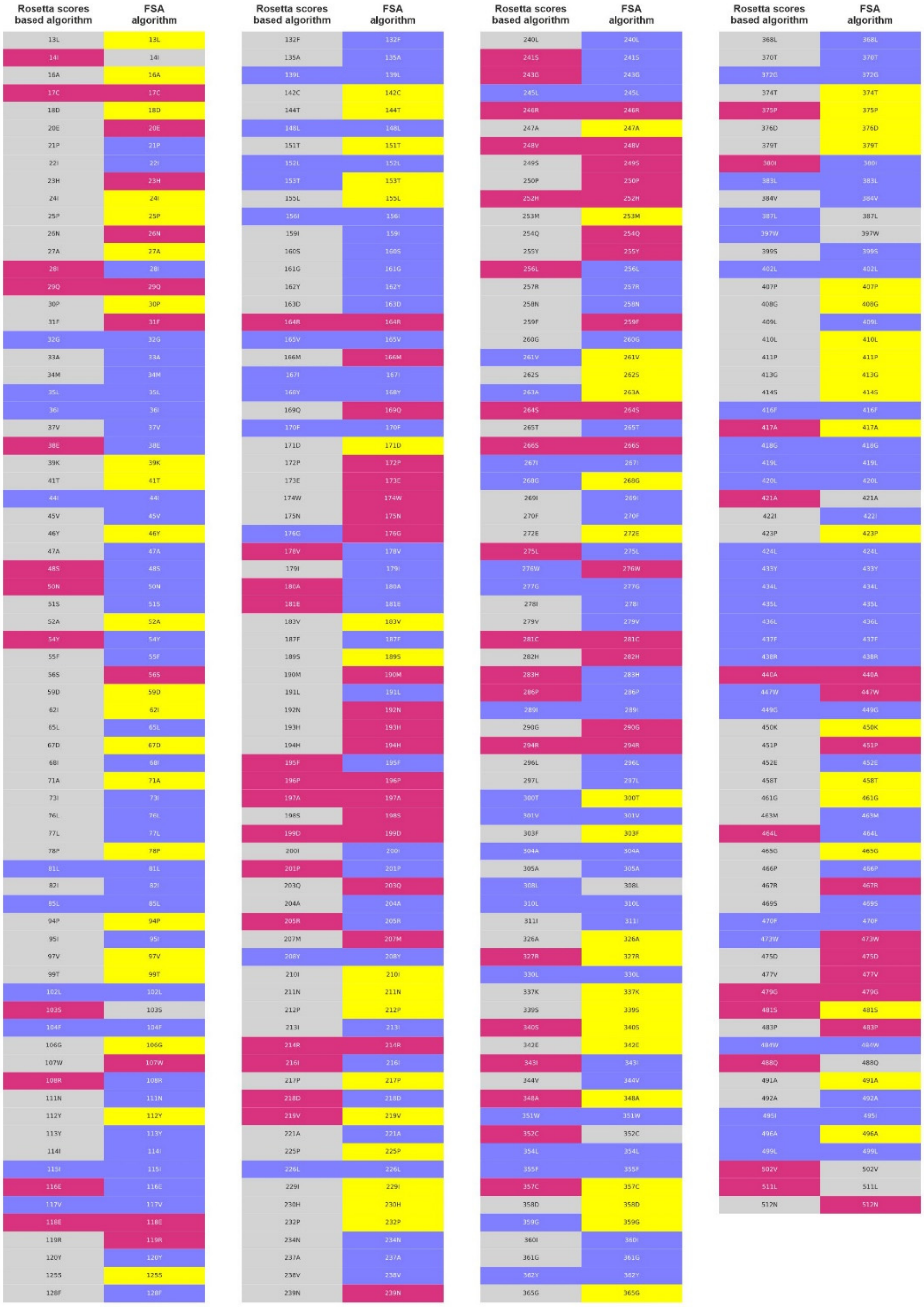

Figure S11: **Annotation of *Is*PadC residues according to the pipeline published by Cagiada et al. versus the FSA pipeline,** related to Figure 4. The FSA pipeline was run as outlined in the main text whereas the other tool was launched with standard settings from a Colab notebook accessible at https://github.com/KULL-Centre/_2022_functional-sites-cagiada. *Is*PadC residues flagged in either or both of the pipelines are displayed with a color code showing the annotated class. Functional (pink) and structural (metallic blue) are assigned by both pipelines whereas adaptability (yellow) is only assigned by the FSA approach.

Table S1: Primers used for the generation of *Is*PadC PSM variants discussed in the main text; related to STAR methods.

Variant	Sequence
IsPSM_D171L_fwd	See IsPSM_E173A_fwd
IsPSM_D171L_rev	AACACGACCATTCCATTCCGGCAAAAACTGATAGATCATCACGCGATCATAACC
IsPSM_P172A_fwd	ATCTATCAGTTTGATGCGGAATGGAATGGTCGTGTTATTGCAGAAAGCGTTC
IsPSM_P172A_rev	ATCAAACTGATAGATCATCACGCGATCATAACCGC
IsPSM_E173A_fwd	TGGAATGGTCGTGTTATTGCAGAAAGCGTTCGTCAGC
IsPSM_E173A_rev	AACACGACCATTCCATGCCGGATCAAACTGATAGATCATCACG
IsPSM_W174G_fwd	ATCTATCAGTTTGATCCGGAAGGTAATGGTCGTGTTATTGCAGAAAGCGTTC
IsPSM_W174G_rev	See IsPSM_P172A_rev
IsPSM_N175A_fwd	ATCTATCAGTTTGATCCGGAATGGGCTGGTCGTGTTATTGCAGAAAGCGTTC
IsPSM_N175A_rev	See IsPSM_P172A_rev
IsPSM_R177Q_fwd	AGCGTTCGTCAGCTGTTTACCAGCATGCTGAATCATCATTTTCC
IsPSM_R177Q_rev	CAGCTGACGAACGCTTTCTGCAATAACCTGACCATTCCATTCCGGATCAAACTG
IsPSM_M190Y_fwd	ACCAGCTATCTGAATCATCATTTTCCGGCAAGCGATATTC
IsPSM_M190Y_rev	ATTCAGATAGCTGGTAAACAGCTGACGAACGCTTTCTG
IsPSM_F259M_fwd	GGTGTTAGCGCAAGCACCAGCATTGGCATTTTTAACGAAG
IsPSM_F259M_rev	GCTTGCGCTAACACCCATATTACGCAGATACTGCATGTGCAGAGG

Table S2: Details of all acquired HDX datasets according to conventions of the HDX-MS community; related to STAR methods.

	**IsPadC PSM wild-type**		**W174G**		**N175A**	
Light conditions	Dark	Light	Dark	Light	Dark	Light
HDX reaction details	10 mM HEPES, 150 mM NaCl, 2 mM $MgCl_2$, pD=7.0, 20 °C					
HDX time course (s)	10, 45, 180, 900, 3600					
HDX control samples	Unlabeled control (wt dark)		Unlabeled control (W174G dark)		Unlabeled control (N175A dark)	
Back-exchange	Not measured					
# of Peptides	63	64	72	73	80	81
Sequence coverage	76%	76%	83%	83%	83%	83%
Avg peptide length / Avg. redundancy	12/1.5	12/1.5	13/1.8	13/1.8	13/1.9	13/1.9
Replicates	3	3	3	3	3	3
Repeatability (average SD for each time point)	0.06, 0.06, 0.13, 0.15, 0.12	0.08, 0.10, 0.11, 0.13, 0.06	0.11, 0.16, 0.19, 0.16, 0.14	0.06, 0.05, 0.03, 0.05, 0.04	0.13, 0.06, 0.05, 0.04, 0.07	0.11, 0.08, 0.11, 0.17, 0.14
Significant differences in HDX	ΔHDX > 0.3 D					
